# Visuospatial imagery-competing task interventions for reducing intrusive memories following real-world trauma: a systematic review and meta-analysis

**DOI:** 10.3389/fpsyt.2026.1925949

**Published:** 2026-10-13

**Authors:** Moayed A. Allahim, Sultan A. Alsallal, Ibrahim A. Almohaimeed, Abdulmajeed A. Alfouzan, Ali S. Alrashed, Rena Y. Abualjamal, Mohammed F. Alharbi, Abdullah N. Alqifari

**Affiliations:** 1 College of Medicine, Qassim University, Buraydah, Saudi Arabia; 2 College of Medicine, King Saud bin Abdulaziz University for Health Sciences, Jeddah, Saudi Arabia; 3 Department of Psychiatry, Eradah Mental Health Hospital, Buraydah, Saudi Arabia; 4 Department of Psychiatry, College of Medicine, Qassim University, Buraydah, Saudi Arabia

**Keywords:** imagery-competing task intervention, intrusive memories, memory reactivation, meta-analysis, post-traumatic stress disorder, systematic review, Tetris

## Abstract

**Background:**

Intrusive memories are a hallmark of PTSD and predict its course, making the early post-trauma period a critical window for prevention. Visuospatial imagery-competing task interventions (ICTI) — brief Tetris-like gameplay after a memory reactivation cue — are grounded in the working memory interference hypothesis and show strong laboratory effects. The only prior RCT synthesis in real-world trauma identified three eligible studies, all rated low certainty. No pre-registered review has synthesised RCT evidence for visuospatial ICTI exclusively in real-world trauma using intrusive memory frequency as the primary outcome.

**Objectives:**

To estimate the pooled effect of visuospatial ICTI on intrusive memory frequency in real-world trauma; examine whether an explicit memory reactivation cue moderates this effect; evaluate effects on PTSD severity, depression, anxiety, adverse events, and attrition; and identify gaps to inform future trial design.

**Methods:**

Seven databases were searched from inception to March 2026 for RCTs of visuospatial ICTI in adults exposed to real-world DSM-5 Criterion A trauma. The primary outcome was intrusive memory frequency via prospective daily diary. Risk of bias was assessed with RoB 2 and ROBINS-I, certainty with GRADE. Random-effects meta-analysis pooled raw mean differences for parallel-group RCTs with compatible diary data. Registered on PROSPERO (CRD420261336501); reported per PRISMA 2020.

**Results:**

Of 818 records identified, 13 studies (9 RCTs, 4 non-randomised) met eligibility criteria. Five parallel-group RCTs (n=359) were pooled for the primary outcome: ICTI reduced intrusive memories by 6.98 fewer episodes/week versus control (95% CI −9.84 to −4.13; I^2^ = 21.9%), moderate-certainty evidence (downgraded for risk of bias). PTSD severity also favoured ICTI (MD −2.42, 95% CI −4.40 to −0.44), low certainty due to inconsistency and indirectness. Depression and anxiety could not be meta-analysed due to measurement heterogeneity. No serious adverse events were attributed to ICTI; attrition was generally low. Non-pooled evidence was broadly consistent, though one VR-based trial and one crossover trial in chronic PTSD found no benefit.

**Conclusions:**

Visuospatial ICTI produces a clinically interpretable, moderate-certainty reduction in intrusive memory frequency after real-world trauma, with low-certainty but favourable effects on PTSD severity. The evidence base remains limited to five pooled RCTs. ICTI should be considered a promising targeted intervention rather than an established treatment, pending larger independent trials.

**Systematic review registration:**

https://www.crd.york.ac.uk/PROSPERO/view/CRD420261336501, identifier CRD420261336501.

## Introduction

Intrusive memories are involuntary, sensory-perceptual re-experiencing phenomena that represent a hallmark symptom of Post-Traumatic Stress Disorder (PTSD) and occur transdiagnostically across depression, anxiety, and other stress-related conditions following real-world trauma (1). Psychological trauma affects approximately 50% of the global population at least once during their lifetime, and the frequency and vividness of intrusive memories in the days and weeks following exposure predict the subsequent course of PTSD identifying the early post-trauma period as a critical and time-limited window for preventive intervention (2, 3).

Visuospatial imagery-competing task interventions (ICTIs) most commonly, brief Tetris-like gameplay administered following a memory reactivation cue are grounded in the working memory interference hypothesis. It is hypothesized that intrusive memories, which are primarily visual-perceptual in character, vie with Tetris-like tasks for the same restricted visuospatial working memory resources; using those shared resources interferes with memory consolidation and subsequent involuntary re-activation (4). This mechanism is supported by independent convergent evidence: a meta-analysis of eye movement paradigms reported an effect size of *d* = 0.74 in laboratory studies (5); a large laboratory meta-analysis of 131 methods across 12,074 participants identified Trauma Reminder and Tetris as the most effective technique (Hedges’ *g* = 0.80) (6); and a further meta-analysis identified visuospatial ICTI as the only cognitive intervention category demonstrating significant effects on intrusion frequency (*g* = 0.46) (7).

Trauma-related mental health conditions represent a substantial and growing global public health burden. The lifetime prevalence of PTSD ranges from 3.9% to 8.8% in high-income countries, rising to 20–30% or above amongst populations exposed to armed conflict, mass casualty events, and acute medical emergencies (2, 8). Intrusive re-experiencing including flashbacks and unwanted trauma memories is not only diagnostically central but is consistently ranked amongst the most distressing and functionally impairing PTSD features, and is associated with elevated rates of comorbid major depressive disorder, substance use disorders, and suicidality (9). As a result, there is a significant financial burden on health systems across the globe, including higher healthcare utilization, lower occupational productivity, and significant indirect expenses (10).

Current guideline-recommended treatments for PTSD including Trauma-Focused Cognitive Behavioural Therapy (TF-CBT), Eye Movement Desensitisation and Reprocessing (EMDR), and Prolonged Exposure (PE) have demonstrated robust efficacy in reducing overall PTSD symptom burden in randomised controlled trials (11, 12). However, single-session psychological debriefing, which was once commonly used in the immediate post-trauma situation, may worsen results for certain people, and the majority of early psychological therapies have failed to prevent the development of PTSD (13). These limitations, combined with the intensity of trauma-focused therapies, high dropout rates (estimated at 20–35% in clinical trials), restricted access in low-resource settings, and the absence of interventions specifically targeting intrusive memory consolidation, have driven substantial interest in brief, scalable, mechanistically grounded early interventions (14): Whilst pharmacological approaches, such as selective serotonin reuptake inhibitors (SSRIs), offer some symptom alleviation, they do not directly address intrusive imagery and pose serious tolerability issues in certain patient populations (12).

Despite a strong laboratory foundation, the only existing RCT-based synthesis of visuospatial ICTI in real-world trauma populations Astill Wright et al. (15) identified only three eligible studies, rated all evidence as low certainty on GRADE, applied a primary outcome (PTSD severity) for which those studies were not powered, and conducted no publication bias assessment (15). Since that review, several additional RCTs have been published in real-world trauma settings (16–21), including a Bayesian adaptive trial in *The Lancet Psychiatry* reporting a marked reduction in intrusive memories (21). However, findings across trials remain heterogeneous, highlighting the need for rigorous synthesis.

To our knowledge no pre-registered systematic review has synthesised RCT evidence for visuospatial ICTI exclusively in real world trauma populations using intrusive memory frequency as the primary outcome. The evidence base for a widely discussed, theoretically grounded, and clinically promising intervention remains thin, methodologically limited, and entirely unsynthesised at the RCT level. Rigorous synthesis of the available evidence is precisely what the field requires before clinical recommendations can responsibly be made.

The present systematic review and meta-analysis aims to synthesize RCT evidence evaluating visuospatial imagery-competing task interventions (ICTIs) for reducing intrusive memories following real-world trauma. Specifically, this review aims to: (1) estimate the pooled effect of visuospatial ICTIs on intrusive memory frequency compared with control conditions; (2) examine whether the presence of an explicit memory reactivation cue moderates intervention effects on intrusive memory frequency; (3) evaluate the effects of visuospatial ICTIs compared with control conditions on PTSD symptom severity and related psychological outcomes, including depression, anxiety, adverse events, and attrition; and (4) identify key gaps in the current evidence base and provide recommendations for future randomized trial design.

## Methods

This systematic review and meta-analysis was conducted and reported in accordance with the Preferred Reporting Items for Systematic Reviews and Meta-Analyses (PRISMA 2020) guidelines and the PRISMA-P checklist for systematic review protocols (22, 23). The review protocol was prospectively registered in the International Prospective Register of Systematic Reviews (PROSPERO) under registration number (CRD420261336501).

### Eligibility criteria

#### Study design

Only randomised controlled trials (RCTs) were eligible for quantitative meta-analysis, encompassing parallel-group RCTs and Bayesian adaptive RCTs. Crossover RCTs were included but analysed separately in a sensitivity analysis given the inherent risk of carry-over effects in memory consolidation paradigms.

Non-randomised studies (feasibility studies, single-group pre-post studies, and single-case experimental designs) were eligible for inclusion in a qualitative narrative synthesis only, to provide contextual evidence on intervention feasibility, acceptability, and effect direction in populations underrepresented in RCTs.

#### Population

Eligible studies enrolled adults aged 18 years and over who had been exposed to real-world trauma as defined by DSM-5 Criterion A: actual or threatened death, serious injury, or sexual violence (1). Exposure was eligible whether direct, witnessed, or occupational (e.g., emergency healthcare workers, first responders). Studies using laboratory analogue trauma (e.g., the distressing film paradigm) in healthy volunteers were excluded.

#### Intervention

Any intervention classified as a visuospatial imagery-competing task was eligible, defined as a task requiring active engagement of visuospatial working memory with the explicit intention of interfering with traumatic memory consolidation or reconsolidation. Eligible interventions included Tetris-based gameplay (with or without mental rotation instructions) delivered via smartphone, tablet, computer, or handheld game console, and other visuospatial tasks delivered via virtual reality.

The following were excluded: verbal cognitive tasks (e.g., word games, reading), eye movement-only interventions (EMDR), pharmacological agents, mindfulness or relaxation-only interventions, and multicomponent interventions in which the visuospatial task could not be isolated as the active ingredient.

Interventions with or without an explicit memory reactivation cue prior to the task were both eligible; the presence or absence of such a cue was coded and examined as a pre-specified moderator.

#### Comparator

Any comparator was eligible, including: passive control (usual care, no intervention, waiting list); active attention-placebo control (e.g., written activity log, podcast, classical music); or an alternative psychological intervention. Comparator type was coded and examined as a pre-specified moderator.

#### Outcomes

The primary outcome was the frequency of intrusive memories (flashbacks, unwanted memories of the trauma) measured via prospective daily diary count of intrusive memory episodes per week. For inclusion in the quantitative meta-analysis, intrusive memory frequency was required to be reported as a per-week count with extractable means and standard deviations to permit raw mean difference pooling. Studies measuring intrusion symptoms via retrospective self-report questionnaire subscales (e.g., PSS intrusion subscale, PCL-5 Re-experiencing subscale) rather than as a daily diary count were eligible for the qualitative narrative synthesis but not for the primary quantitative meta-analysis. Intrusive memory frequency was extracted at each trial’s own pre-specified primary diary timepoint (range: Week 1 to 6 weeks post-intervention) to maximise trial inclusion and provide the most comprehensive estimate of overall intervention effectiveness.

Secondary outcomes comprised:

PTSD symptom severity: total score on a validated measure (PCL-5, IES-R, ASDS, or PSS) at the earliest available timepoint post-intervention.

Depression: total score on a validated measure (HADS-D, PHQ-2, EPDS) at the earliest available timepoint post-intervention.

Anxiety: total score on a validated measure (HADS-A, GAD-2) at the earliest available timepoint post-intervention.

Adverse events: any reported adverse events, distress responses, or symptom exacerbation attributed to the intervention.

#### Language and date restrictions

No language restrictions were applied at the search stage. Searches were conducted from database inception to March 2026, with no date restriction. All identified records meeting the eligibility criteria were published in English.

### Information sources and search strategy

We systematically searched seven electronic databases from inception to March 2026: PubMed, MEDLINE, Scopus, Web of Science, Psychology Collection (ProQuest Psychology Journals), Google Scholar, and Ovid. No language or date restrictions were imposed at the search stage.

A core search string combining intervention, outcome, and study-design concepts was developed and then adapted to the syntax of each database. A representative version was as follows:

(“Tetris” OR “visuospatial task” OR “imagery-competing task” OR “cognitive task intervention” OR “visuospatial interference” OR “mental rotation” OR “spatial task” OR “ICTI”) AND (“intrusive memor*” OR “intrusion*” OR “flashback*” OR “re-experiencing” OR “PTSD” OR “post-traumatic stress”) AND (“randomized controlled trial” OR “randomised controlled trial” OR “RCT” OR “random*”). To increase sensitivity, we added trauma-specific terms (for example, “emergency department”, “road traffic accident”, “caesarean section”, and “healthcare worker”) in targeted supplementary searches. For Google Scholar, the first 100 records returned by the targeted string were screened by title and abstract, and potentially eligible reports were added manually to the screening library. The full, database-specific search strategies are reported in **Supplementary File S1**.

### Study selection

All records were imported into Rayyan systematic review software and deduplicated prior to screening (24). Study selection proceeded in two sequential stages.

In the first stage, two independent reviewers (IRs) screened all titles and abstracts against the eligibility criteria. Records were excluded at this stage only where they clearly did not meet all PICOS criteria. Disagreements were resolved by discussion; a third reviewer arbitrated where consensus could not be reached.

In the second stage, all records passing title and abstract screening were retrieved in full text. Both IRs independently applied the full eligibility criteria to each full-text record. Reasons for exclusion at the full-text stage were documented and are reported in the PRISMA 2020 flow diagram.

### Data extraction

Data were extracted independently by two reviewers using a standardised extraction form piloted on the first three included studies prior to full extraction; amendments to the form were documented. Disagreements were resolved by third-party arbitration. Corresponding authors were contacted for missing or unclear data where applicable.

The following data were extracted for each included study: Study characteristics: authors, year, country, journal, study design, and sample size. Population characteristics: trauma type and setting, time since trauma at enrolment, age, sex/gender, clinical characteristics, and presence of pre-existing PTSD diagnosis. Intervention characteristics: task type, presence and nature of memory reactivation cue, session duration and number, total dose, timing relative to trauma (hours, days, weeks), delivery mode, and adherence/compliance rates. Comparator characteristics: type, content, and delivery. Outcome data: for all primary and secondary outcomes at all available timepoints, including adverse events and attrition.

### Risk of bias assessment

Risk of bias in individual randomised studies was assessed independently by two reviewers using the Cochrane Risk of Bias 2 (RoB 2) tool for randomised controlled trials (25). RoB 2 evaluates five domains: bias arising from the randomisation process, bias due to deviations from intended interventions, bias due to missing outcome data, bias in measurement of the outcome, and bias in selection of the reported result. Each domain was rated as low risk, some concerns, or high risk, and an overall risk of bias judgement was derived for each study.

For the non-randomised studies included in the qualitative narrative synthesis, risk of bias was assessed using the ROBINS-I tool (26). ROBINS-I evaluates seven domains: confounding, participant selection, intervention classification, deviations from intended interventions, missing data, outcome measurement, and selective reporting. Each domain was rated as low, moderate, serious, or critical risk, and an overall judgement was derived for each study.

Disagreements were resolved through discussion, with a third reviewer arbitrating when consensus was not reached. Risk of bias assessments are presented visually using traffic light plots and summary bar charts.

### Data synthesis and statistical analysis

#### Quantitative synthesis

Where two or more studies reported the same outcome with sufficient data, random-effects meta-analysis was conducted using the DerSimonian–Laird estimator for between-study variance (τ^2^) (27). All analyses were conducted using OpenMetaAnalyst (28).

For the primary outcome, intrusive memory frequency reported as daily diary counts per week, raw mean differences were calculated as the mean difference between the ICTI and control groups. Raw mean differences were selected because they provide clinically interpretable effect estimates in the original unit of measurement, namely intrusive memories per week. Negative values indicate fewer intrusive memories in the ICTI group compared with the control group.

A metric sensitivity analysis comparing Hedges’ g and log-incidence rate ratio pools using subsets of trials with compatible data formats is reported in **Supplementary File S1**. This analysis was conducted to examine whether the choice of effect size metric influenced the primary conclusion.

#### Primary meta-analysis

The primary meta-analysis pooled five parallel-group RCTs reporting intrusive memory frequency as a daily diary count per week [Iyadurai (38), Horsch (39), Kanstrup (36), Iyadurai (17), Kanstrup (19)], using each trial’s own pre-specified primary diary timepoint (range: Week 1 to Week 5 post-intervention).

Three eligible parallel-group RCTs were excluded from the primary pool because their data formats were incompatible with raw mean difference pooling and are reported narratively in the qualitative synthesis. The crossover trial (Kehyayan (20)) was analysed separately as a standalone sensitivity analysis.

#### Assessment of heterogeneity

Statistical heterogeneity was assessed using the I^2^ statistic and Cochran’s Q test (29). I^2^ values were interpreted as follows: 0–25% indicating low heterogeneity, 26–50% moderate heterogeneity, 51–75% substantial heterogeneity, and greater than 75% considerable heterogeneity. When I^2^ exceeded 50%, potential sources of heterogeneity were explored through pre-specified subgroup analyses.

#### Pre-specified subgroup analyses

The presence or absence of an explicit memory reactivation cue before the visuospatial task was pre-specified as the primary moderator hypothesis, based on reconsolidation theory and the proposed mechanism of ICTIs. Studies using an explicit memory reactivation cue were hypothesised to show larger effects than studies without such a cue. Subgroup pooled estimates and tests for subgroup differences are reported in the Results.

#### Publication bias

Publication bias was explored through visual inspection of funnel plot asymmetry. Formal statistical tests for funnel plot asymmetry, including Egger’s regression test, were not performed because fewer than 10 studies contributed to the pooled analysis, in accordance with Cochrane Handbook guidance (30, 31). Therefore, the funnel plot was interpreted descriptively, and no definitive conclusions regarding the presence or absence of publication bias were drawn from this small set of studies.

#### GRADE assessment

The certainty of evidence for each outcome was assessed using the GRADE framework (32). For outcomes where meta-analysis was not performed, including depression and anxiety, certainty was graded narratively based on risk of bias, inconsistency, indirectness, imprecision, and publication bias.

## Results

A total of 818 records were identified across seven electronic databases (**Figure 1**). After removal of 186 duplicate records, 632 records were screened by title and abstract, of which 608 were excluded as irrelevant. Twenty-four reports were retrieved and assessed for full-text eligibility, of which 11 were excluded with documented reasons. Thirteen studies met the eligibility criteria for inclusion in the systematic review: 9 randomised controlled trials and 4 non-randomised studies. Five parallel-group RCTs were included in the quantitative meta-analysis of intrusive memory frequency. Eight studies were included in qualitative narrative synthesis only: four RCTs whose data formats were incompatible with raw mean difference pooling and four non-randomised studies.

**Figure 1:**
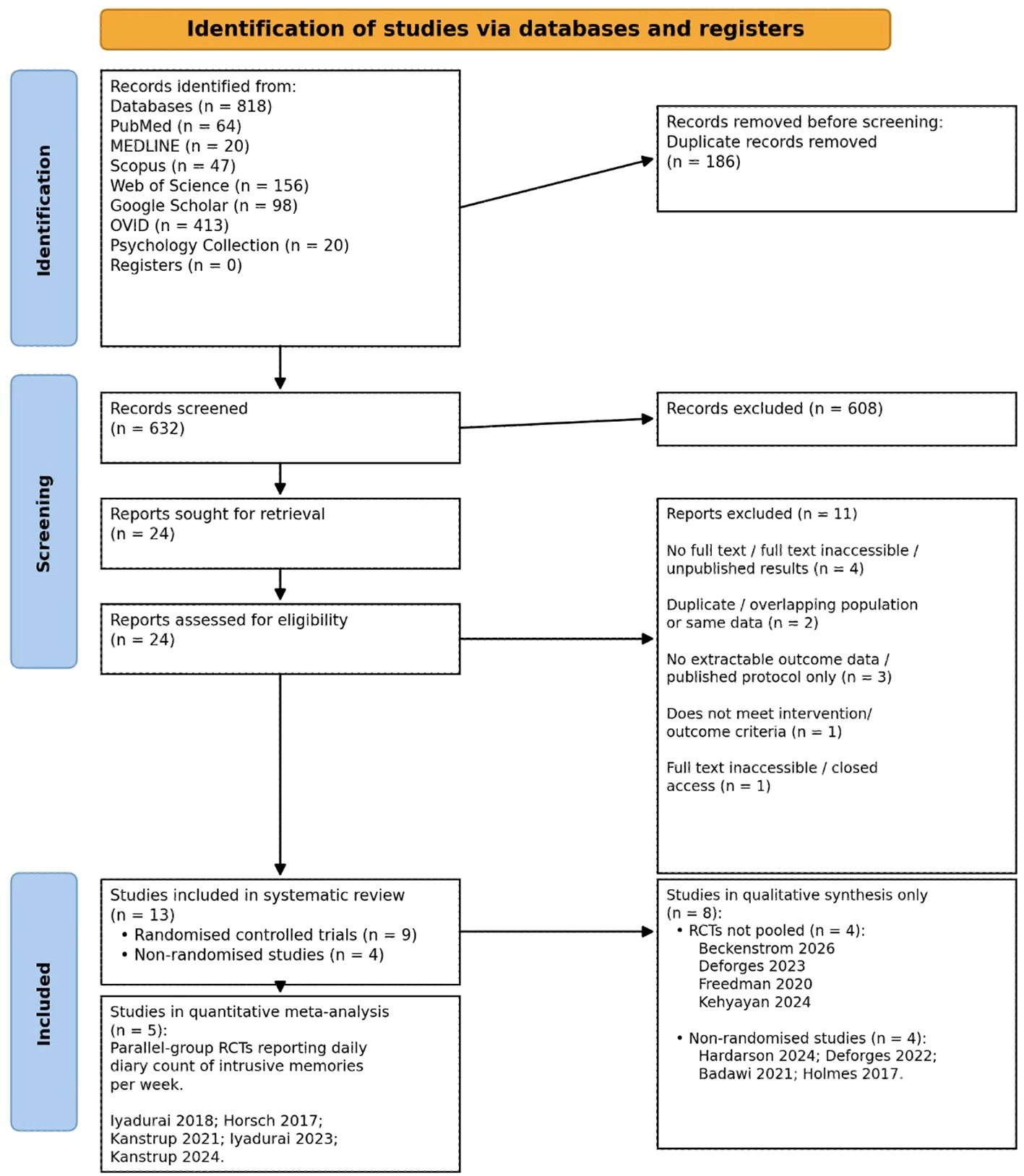
PRISMA 2020 flow diagram for the systematic review of visuospatial imagery-competing task interventions (ICTI) for intrusive memories following real-world trauma.

The table includes 13 studies published between 2017 and 2026 **Table 1**. Designs range from RCTs (parallel, crossover, adaptive) to feasibility and pre-post studies. Recruitment settings include emergency departments, maternity units, intensive care units, refugee centers, and inpatient psychiatric hospitals. Sample sizes vary: largest randomized sample = 146, smallest feasibility = 12. Mean age between 22 years (young refugees) and 46 years (ED trauma patients). Gender distribution is predominantly female (up to 100% in childbirth studies; the lowest female proportion was in Freedman (37), with 21/77 female participants, approximately **27.3%**. Trauma populations include healthcare workers (COVID-19), refugees, motor vehicle accident survivors, women after emergency cesarean section, and chronic PTSD patients. Baseline intrusive memory frequency is reported in most studies as diary counts: e.g., median 14–15/week in healthcare staff, median 15/week in Kanstrup (19), and mean ~2/day in long-standing trauma. Time since trauma ranges from <6 hours (acute) to >20 years. Follow-up from 1 week to 6 months. Attrition rates: up to 35% in Beckenstrom (ICTI arm) and 33% in Hardarson (feasibility); many studies report low or no differential dropout.

**Table 1:** Study and participant characteristics across 13 included studies.

First author, year	Study design	Recruitment setting	Sample size randomized	Sample size analyzed	Mean age ± SD	Gender (M/F/Other)	Trauma population	Trauma type	Baseline PTSD symptoms	Baseline intrusive memory frequency	Time since trauma at enrollment	Follow-up duration	Timepoints assessed	Attrition rate	Loss to follow-up reasons (if reported)
Beckenstrom et al., 2026 (21)	Decentralized, parallel-group, Bayesian adaptive randomized controlled trial (RCT)	UK NHS facilities admitting COVID-19 patients; recruited via Intensive Care Society networks, social media, seminars, posters	Total: 99; ICTI: 40, Active Control: 39, Treatment as Usual (TAU): 20	Primary outcome (week 4): ICTI = 26, Active Control=31, TAU = 19 (ITT analysis)	41.2 ± 10.2 years	12/85/2	Healthcare staff (NHS) exposed to work-related trauma during the COVID-19 pandemic	Work-related traumatic events meeting DSM-5 Criterion A	PCL-5 (20-item version); 49% met probable PTSD threshold (PCL-5 ≥33) at baseline. Per-arm means and SDs available on OSF (osf.io/cs6hn) but NR in the main paper.	Inclusion required ≥3 intrusive memories of the past week. Combined baseline median 10.0/week (IQR 6.5–17.5). By arm: ICTI 11.5/week (IQR 9.0–18.0); Active Control 9.0 (IQR 6.5–16.0); TAU 6.5 (IQR 5.0–17.75).	Variable; from day of trauma to months later (“6-month post-trauma window”)	24 weeks	Baseline, week 4 (primary), week 12, week 24	ICTI 14/40 (35%), Active Control 8/39 (21%), TAU 1/20 (5%). Overall retention is lower in ICTI vs TAU (BF = 8.12).	Withdrawals due to adverse events (n=3; diary burden n=2, unrelated SAE n=1), insufficient time (n=1), loss to follow-up, or incomplete diaries.
Kanstrup et al., 2024 (19)	Parallel-group, two-arm RCT	Healthcare facilities in Sweden, social media, hospital resources, and newspapers	Total 144; Intervention=73, Control=71	ITT: 144; primary outcome completers: 118 (Control 58, Intervention 60)	41.41 ± 10.89	25/118/1	Healthcare workers in Sweden during the COVID-19 pandemic	Work-related traumatic events	PCL-5 (8-item): mean 12.10 ± 6.63 (scale 0-32; cutoff for probable PTSD typically 19)	Median 15.0 (IQR 6.25–28.75) per week (baseline diary).	72.2% within 1–3 months; others within 24h to >3 months	6 months	Baseline, week 1, week 5 (primary), 1 month, 3 months, 6 months	6.3% overall (9/144 discontinued; 13 lost to follow-up). Intervention: 8 lost, 5 discontinued; Control: 5 lost, 4 discontinued	NR
Kehyayan et al., 2024 (20)	Randomized crossover trial (within-patient)	Outpatient unit, Department of Psychosomatic Medicine and Psychotherapy, LWL-University Hospital, Bochum, Germany	Total 38 (all received both interventions in crossover order)	38 (ITT; all who submitted diary data and were randomized)	42.2 ± 13.3	7/31	PTSD (ICD-10 F43.1; 89.5%) or other reactions to severe stress (ICD-10 F43.8; 10.5%)	Type I (single event) 34.2%, Type II (complex) 65.8%	DSM-5 PTSD diagnosis (CAPS not reported; clinical diagnosis). Comorbid depression 81.6%, anxiety 42.1%	~14.1/week (raw: 2.02/day, SD 1.92)	Mean 13.8 years (SD 15.0) since traumatic event(s)	8 weeks	Baseline (2-week diary), T1 (week 2, first intervention), T2 (week 4, second intervention), T3 (week 8, final diary)	30/68 recruited excluded (did not meet criteria, withdrew, or had <3 intrusions/week). Of 38 analyzed, all completed per protocol (crossover design)	NR
Hardarson et al., 2024 (33)	Feasibility study (repeated AB design, pre-post)	Sub-study of SAGA cohort (population-based longitudinal study of Icelandic women); remote recruitment	Total enrolled = 12 (all female); no randomisation (single group)	Primary outcome (Week 5): n=8; 3-month follow-up: n=7	42.42 ± 12.03	All female (100%)	Trauma-exposed women (community sample) with long-standing intrusive memories	Sexual violence (n=5), witness to death/serious injury (n=3), physical violence (n=3), motor vehicle accident (n=1)	PCL-5 at baseline: mean 36.42 (SD 16.81) – moderate-severe range	Mean 25.98 (SD 29.39) per week (baseline diary)	Mean 20.73 years (SD 14.65) since target trauma	3 months after the second intervention session	Baseline (Week -1), Week 0 (intervention sessions), Weeks 1–4, Week 5 (primary), 3-month follow-up	4/12 (33%) did not complete the primary outcome: 2 completed Week 1 but lost contact, 2 dropped out before Week 1 due to unrelated stressors	Unrelated stressors, unable to contact
Deforges et al., 2023 (16)	Two-arm, parallel-group, multicentre, double-blind superiority RCT	Two Swiss University Hospital maternity units (Lausanne and Geneva)	Total 146; Intervention (Tetris) = 74, Attention-placebo control = 72	PCL-5 at 6 weeks: 122 (Intervention 64, Control 58); CAPS-5 at 6 weeks: 116 (Intervention 62, Control 54)	33.9 ± 4.2	All female (100%)	Women after unplanned cesarean section (UCS) at ≥34 weeks of gestation, live birth, perceived childbirth as traumatic	Childbirth-related trauma (unplanned cesarean section, DSM-5 Criterion A event)	PCL-5 (20-item) collected at baseline; per-arm means NR.	PCL-5 (20-item) administered at baseline within 6 hours postpartum per study protocol; per-arm baseline means and SDs not reported in publication. Week 1 daily diary collected; per-arm Week 1 means NR in publication.	Within 6 hours after an unplanned cesarean section	6 months	Baseline (within 6h), 1st week diary, 6 weeks (primary), 6 months	At 6 weeks, PCL-5: 24/146 (16.4%) missing. Intervention: 10 lost (3 no intervention, 3 dropped T1-T2, 4 T3-T4); Control: 14 lost (2 no task, 5 dropped T1-T2, 7 T3-T4)	Withdrawal, loss to follow-up (NS); COVID-19 pandemic pause
Iyadurai et al., 2023 (17)	Two-arm, parallel-group, single-blind RCT with delayed-access comparator (active intervention offered to comparator arm after Week 4 primary endpoint for ethical reasons)	UK NHS Intensive Care Units (ICUs) during the COVID-19 pandemic; recruited via Intensive Care Society networks	Total 86; Immediate intervention = 43, Delayed (comparator) = 43	ITT: 86 (primary outcome: 75 with full/partial data). Per-protocol: 76	38.7 ± 9.9	15/69/2	ICU staff (nurses, doctors, allied health) working clinically during COVID-19	Work-related traumatic events meeting DSM-5 Criterion A	PCL-5 (4-item version) at baseline: Immediate 8.71 ± 3.64, Delayed 8.00 ± 3.27. Also IES-R total	Median 14 (IQR 9–20) per week (baseline diary)	Mixed: 8.3% within 24h, 19.1% within 1 month, 16.7% 1–3 months, 54.8% >3 months; ongoing exposure reported by >88%	8 weeks (delayed arm received intervention at week 4, followed for another 4 weeks)	Baseline, week 4 (primary), week 8	Overall attrition at week 4: 11/86 (12.8%). Immediate: 7/43 (16.3%) missing primary outcome; Delayed: 4/43 (9.3%) missing. No withdrawals due to AEs	NR
Deforges et al., 2022 (34)	Proof-of-principle pre-post study (single group)	Flyers at Lausanne University Hospital (Switzerland) and contact from previous observational studies	Total enrolled = 18 (no randomization; single intervention group)	Primary outcome (diary): n=18	33.55 ± 6.35	All female (100%)	Women who had given birth at Lausanne University Hospital ≥6 weeks prior, reporting ≥4 childbirth-related intrusive memories (CB-IMs) in the past 2 weeks.	Traumatic childbirth	PCL-5 (CB-PTSD) pre-intervention: mean 27.89 (SD 12.14); 8/17 (47%) met CB-PTSD diagnostic criteria	Median 11 (IQR 6) per 2 weeks (diary 1 = 14 days); approximately 5.5 per week	Median 2.01 years (IQR 2.23); range 7 months to 6.9 years	6 weeks post-intervention (diary 3 up to week 6)	Baseline (diary 1: 14 days), intervention day, diary 2 (first 14 post-intervention days), diary 3 (days 28-42, i.e., weeks 5-6), 1-month PCL-5	0% post-intervention (no dropouts after intervention); 1 participant excluded from primary analysis due to non-compliance with intervention instructions	Non-compliance (P18 did not follow instructions; her data were excluded from primary outcome analysis)
Badawi et al., 2021 (35)	Feasibility study (single-group, pre-post, adjunctive)	Inpatient psychiatric hospital (PTSD treatment programme, Australia)	Total enrolled = 23 (all received treatment-as-usual + adjunctive Tetris on 3 occasions); no randomisation	Primary feasibility outcomes: n=15 (completed protocol). Efficacy analyses: n=9–10 for some measures	NR	NR	Adults with PTSD (CAPS-5 diagnosed) attending inpatient treatment	Mixed trauma (military combat, first responder duty, occupational injury)	PCL-5 pre-treatment: mean 59.0 (SD 8.0), n=9; IES-R intrusions pre: mean 30.56 (SD 5.36), n=9	NR	NR	21 days (3 weeks)	Day 0 (baseline), days 1–21 (three daily intrusion monitoring), day 21 (post-intervention)	8/23 (34.8%) discontinued before completing 3 Tetris sessions: 5 changed treatment (no imaginal exposure), 3 early discharge	No imaginal exposure in therapy sessions, early discharge
Kanstrup et al., 2021 (36)	Exploratory open-label pilot RCT (parallel-group)	Emergency Department (ED), Karolinska University Hospital Huddinge, Stockholm, Sweden + nearby ED Walk-in Center	Total 41; Intervention (Tetris) = 22, Active control (podcast) = 19	Primary outcome (week 1 diary): Intervention 20, Control 19; Week 5 diary: Intervention 18, Control 18	46.15 ± 15.77	Total 23 females (56%), 18 males (44%); Intervention 14F/8M, Control 9F/10M	Trauma-exposed patients presenting to the ED (mixed trauma)	Transportation accidents (36.8% control, 36.4% intervention), serious accidents at work/home, assault, etc. DSM-5 Criterion A	Not assessed as baseline	Not assessed at baseline	74% within 6 hours; 26% within 72 hours (protocol change)	6 months	Baseline (day 1 in ED), week 1 (primary), week 5 (secondary), 3 months, 6 months	Week 1: 2/22 intervention missing (9%), 0/19 control missing. Week 5: 4/22 intervention (18%), 1/19 control (5%). 6 months: 8/22 intervention (36%), 1/19 control (5%)	NR
Freedman et al., 2020 (37)	Pilot RCT (parallel-group, no blinding of treatment delivery, assessor-blind)	Emergency Department (ED), Level III Trauma Medical Center, Israel	Total 77; SnowWorld VR = 36, Control (no intervention) = 41	2 weeks: 55 (SnowWorld 28, Control 27); 6 months: 49 (SnowWorld 28, Control 21)	SnowWorld 35.36 ± 13.48; Control 34.95 ± 15.47	SnowWorld: 26/10; Control: 30/11	Adult survivors of traumatic events presenting to the ED.	84.1% Motor Vehicle Accidents; other: falls, assaults, work accidents; DSM-IV Criterion A	PSS (Posttraumatic Symptom Scale) at baseline (prior trauma): SnowWorld mean 2.39, Control 2.05 (not clinically elevated)	Not assessed at baseline	Mean <1 hour (85.5% arrived and recruited within 1 hour; all but one within 5 hours; one at 8 hours)	6 months	Baseline (ED), 2 weeks, 6 months	2 weeks: 22/77 (28.6%) participants were missing, balanced between groups. 6 months: 28/77 (36.4%) were missing, with higher attrition in the control group (48.8% vs 22.2%), potentially affecting interpretation of long-term outcomes.	NR
Iyadurai et al., 2018 (38)	Proof-of-concept RCT (parallel-group, single-blind)	Emergency Department, John Radcliffe Hospital, Oxford, UK	Total 71; Intervention (Tetris) = 37, Control (activity log) = 34	ITT primary outcome: 71 (imputed). Diary returned: 67 (Intervention 34, Control 33)	39.66 ± 16.32	34/37	Motor vehicle accident survivors presenting to the ED	Motor vehicle accidents (car, motorcycle, cyclist, pedestrian); DSM-IV Criterion A1	Not assessed at baseline	Not assessed at baseline	Mean 192 min (Intervention) to 211 min (Control) within 6h	1 month	Baseline (ED), daily diary for 1 week (primary), 1 week questionnaires, 1 month questionnaires	1 week: 4/71 (5.6%) missing; 1 month: 9/71 (12.7%) missing. No differential attrition reported	NR
Horsch et al., 2017 (39)	Proof-of-principle RCT (parallel-group, open-label)	Maternity ward, University Hospital Lausanne, Switzerland (post-emergency cesarean section)	Total 56; Intervention (Tetris) = 29, Control (usual care) = 27	ITT: 56 (primary outcome). Per protocol: 49 (Intervention 25, Control 24)	Full sample mean 33.39 ± 4.22; Intervention 34.24 ± 3.81; Control 32.37 ± 4.47	All female (100%)	Women after emergency cesarean section (ECS) with live birth	Emergency cesarean section (traumatic childbirth, DSM-IV Criterion A)	Not assessed	Not assessed	Within 6 hours after ECS	1 month	Baseline (within 6h), daily diary for 1 week (primary), 1 week questionnaires, 1 month questionnaires	ITT: no attrition for primary (imputed). Per protocol: 7 excluded (3 intervention did not play ≥10 min; 4 control excluded for protocol violations). 1 month: some missing, imputed	NR
Holmes et al., 2017 (40)	Feasibility study (single-group, pre-post)	Sweden (refugee accommodation, NGOs, social media, language classes, central station)	Total enrolled = 22 (single group; no randomisation)	Primary feasibility outcomes: n=22; Diary return: n=17 (77%)	22 ± 2.4 (range 16–25)	17/5	Young refugees (16–25 years) recently arrived in Sweden (82% within the last 6 months)	Trauma in the home country or during escape (Syrian conflict); not further specified	Not assessed	~12.65/week (raw: 1.81/day)	Time since traumatic event NR (ongoing conflict, undatable); 82% of participants arrived in Sweden within 6 months of study enrolment.	1 week (diary) plus immediate post-intervention ratings	Baseline (interview), daily diary for 1 week, post-intervention acceptability ratings	5/22 (23%) did not return diary: 1 too sad (news from Syria), 1 did not understand instructions, 3 dropped out without reason	Emotional distress, lack of understanding, and no contact

As shown in **Table 2**, the active intervention (“ICTI”) consistently involves: a) a brief memory reminder cue (e.g., listing a traumatic “hotspot” or briefly recalling the event), followed by b) playing Tetris with mental rotation instructions for 12–25 minutes. Delivery: in-person (tablet/game console) or remote via smartphone/computer (i-spero, SMART-TRIAL). Therapist involvement: guided by researchers (including non-specialists after training) or self-administered after a single guided session. Adherence: generally >90% (e.g., 97% in Beckenstrom, 96% in Kanstrup (19), 100% in Freedman). Comparator types: active controls (podcast listening, activity log, Wikipedia article) and passive controls (treatment-as-usual, delayed intervention, or baseline monitoring). Control tasks are matched for duration and researcher contact but lack visuospatial interference. Feasibility studies used no control group or within-subject baseline. One crossover trial of Kehyayan et al. (20) compared Tetris with a verbal task (Wiki) and a never-targeted memory task.

**Table 2:** Intervention and comparator details of the imagery-competing task.

First author, year	Intervention name	ICTI type	Memory reminder cue (Y/N)	Theoretical model	Timing after trauma	Number of sessions	Session duration (min)	Delivery format	Therapist involvement	Device/platform	Adherence/compliance	Co-interventions	Comparator type	Control task description
Beckenstrom et al., 2026 (21)	Imagery-Competing Task Intervention (ICTI)	Digital, brief, single guided session + optional self-use	Yes (brief recall of target intrusive memory image)	Memory reconsolidation, working memory taxation (visuospatial interference)	Variable (day of trauma to months later)	ICTI: 1 guided + as-needed self-use (mean 6.91 over 24 wk); Active control: same structure (mean 5.57); TAU: 0	Guided session 1 hour; gameplay/listening 20 min per use	Remote, app-based (i-spero platform) for ICTI and active control; TAU: none	Guided by a digital navigator (not required to be a mental health professional) for ICTI and active control; TAU: none	i-spero (smartphone, tablet, computer) for ICTI and active control; TAU: none	ICTI: 33/34 (97%) completed guided session; Active control: 34/35 (97%)	Allowed: at baseline, ICTI 38%, Active control 38%, TAU 50% on psychotherapy/medication	Active control (music-listening) + Treatment as Usual (TAU)	Active control: listen to Mozart’s String Duo No.1 K.423 for 20 min + informational podcast about Mozart. TAU: no task; continued own care, offered access after trial.
Kanstrup et al., 2024 (19)	Imagery-Competing Task Intervention (ICTI) – Tetris with mental rotation	Digital, brief, single guided session + optional self-use	Yes (brief description of one intrusive memory “hotspot”)	Memory reconsolidation, visuospatial interference (working memory taxation)	Variable: from 24 hours to months after trauma	1 guided session (day 1) + as-needed self-use (80.9% used on their own at 1 month)	Task component ~25 min (20 min Tetris gameplay)	Remote, app-based (SMART-TRIAL + external www.tetris.com)	Guided by a researcher (non-specialist) for the initial session, then self-administered	Participants’ own smartphone (access to the Tetris website)	Intervention: 70/73 (96%) completed guided session; Control: 68/71 (96%)	Allowed to continue usual care; no restrictions on mental health treatments	Active control (attention-based placebo)	Listen to the philosophy podcast (Filosofiska Rummet, Sveriges Radio) for 20 min, focusing on ideas and meaning.
Kehyayan et al., 2024 (20)	Visuospatial Tetris-based intervention (memory reminder + Tetris)	Digital, single-session per targeted memory (two sessions total per patient, crossover)	Yes (written report of traumatic memory, then destroyed – memory reminder cue)	Memory reconsolidation (reactivation + updating) and visuospatial interference (dual-task, mental rotation)	Long-standing trauma (mean 13.8 years after event)	One session per targeted memory (two memories targeted per patient, one with Tetris, one with Wiki, in random order)	~35 min total (10 min memory report + 25 min Tetris)	In-person (lab) using a tablet computer	Guided by a researcher (not blind to the intervention)	Samsung Galaxy tablet (10.1-inch) with Tetris app (N3TWORK)	All 38 patients completed both intervention sessions (no attrition within the analysed sample)	NR	Verbal condition (Wiki) + never-targeted memory (no intervention)	Wiki: read a shortened Wikipedia article about postage stamps (~30 min) + answer 8 multiple-choice questions (~5 min). No intervention: no task, only diary monitoring
Hardarson et al., 2024 (33)	Imagery-competing task intervention (Tetris with mental rotation)	Brief, remotely delivered, guided + self-use	Yes (participant briefly brought the intrusive memory to mind without discussing content)	Memory reconsolidation (reactivation + updating) and visuospatial interference (mental rotation)	Long-standing trauma (mean 20.7 years after event)	At least 2 guided sessions (up to 6) plus self-guided use as needed	~20 min of Tetris gameplay per session	Remote (video platform or telephone)	Guided by non-specialist researchers (psychology students) with remote training/supervision, then self-administered	Participant’s own computer/smartphone (www.tetris.com)	8/12 (67%) completed ≥2 guided sessions. Of these: 7 completed exactly 2 sessions, 1 completed 4 sessions. Amongst the 4 who did not complete ≥2 sessions: 1 completed 1 session, 2 completed 0 sessions, 1 unclear from publication.	NR	Baseline phase (no intervention) – within-subject control	No task; only monitoring of intrusive memories in a daily diary for one week
Deforges et al., 2023 (16)	Visuospatial task (Tetris)	Single-session, preventive	No	Visuospatial interference (working memory taxation), memory consolidation disruption	Within 6 hours postpartum	1 session (plus 3 min training)	15 min gameplay (plus 3 min training = 18 min total)	In-person, at the participant’s hospital bed	Delivered by trained maternity clinicians (midwives/nurses) – guided, not self-administered	Handheld gaming device (Nintendo DS)	71/74 (96%) received the intervention (3 did not receive, reasons NS)	Routine postpartum care; no restrictions	Attention-placebo control	Written activity log for 15 min: participant briefly reports nature and duration of activities
Iyadurai et al., 2023 (17)	Imagery-Competing Task Intervention (ICTI) – Tetris with mental rotation	Digital, brief, single guided session + optional self-use	Yes (participants briefly described one intrusive memory “hotspot” from a list)	Memory reconsolidation (reactivation + updating) and visuospatial interference (working memory taxation)	Variable: days to months after trauma (also ongoing trauma)	1 guided session (researcher-led) + as-needed self-use over 4 weeks	Guided session ~1 hour; gameplay 20 min per use	Remote, via secure web platform (i-spero) + Microsoft Teams for guided session	Guided session by trained researchers (clinical psychologists and research assistants); then self-administered	Participant’s own smartphone, tablet, or computer (i-spero platform, Tetris embedded)	Immediate arm: 38/40 (95%) adhered to guided session; Delayed arm: 38/38 (100%) after crossover	Allowed to continue usual care (any treatments they would otherwise access)	Delayed access (usual care for 4 weeks, then same intervention)	No active control task; comparator arm received usual care (no task) for the first 4 weeks, then crossed over to receive the same ICTI
Deforges et al., 2022 (34)	Behavioral intervention (memory reminder + Tetris)	Single-session treatment (long-standing trauma)	Yes (multi-sensory: returning to maternity ward, walking past delivery suite, plus oral narrative of childbirth in chronological order, then specific narrative of most frequent IM)	Memory reconsolidation (reactivation + updating) and visuospatial interference (Tetris with mental rotation)	Years after trauma (median 2 years)	1 session (single-session)	Narrative tasks ~10–12 min, 10-min gap (assumed labilization), 20 min Tetris gameplay	In-person: started in a neutral office, moved to the maternity ward examination room, then back to the office	Guided by a study psychologist (CD) – not self-administered	Handheld gaming device (Nintendo 3DS) with Tetris	17/18 (94%) complied with intervention instructions (1 non-compliant excluded)	None (exclusion criterion: ongoing childbirth-related psychological treatment)	Baseline phase (pre-intervention) – within-subject control	No task; only monitoring of intrusive memories in the daily diary for 14 days pre-intervention
Badawi et al., 2021 (35)	Tetris intervention (visuospatial cognitive task)	Adjunctive, multiple-session (3 occasions)	Yes (imaginal exposure during routine trauma-focused therapy session – at least 15 min – served as reactivation cue)	Memory reconsolidation (reactivation via imaginal exposure + visuospatial interference)	Chronic PTSD (years after index trauma)	3 sessions (each after a therapy session with ≥15 min imaginal exposure)	12 min Tetris gameplay per session (plus mental rotation practice pre-study)	In-person, on the hospital ward, after a therapy session	Clinician provided tablet; participant played self-administered after the trauma-focused therapy session.	Lenovo tablet (10-inch screen) with modified Tetris (UTS Research Version, touch-screen)	15/23 (65%) completed all 3 sessions (of those who started treatment with imaginal exposure)	Treatment-as-usual (trauma-focused CBT with imaginal exposure, 3–4 sessions/week for 3–5 weeks) – same for all participants	Within-subject baseline (pre-intervention monitoring period)	No task; participants monitored intrusive memories via a phone app for 3 days (baseline) before any Tetris session
Kanstrup et al., 2021 (36)	Cognitive task intervention (Tetris with mental rotation)	Single-session, delivered acutely	Yes (participants listed “hotspots” – worst moment images – on a sheet of paper, kept visible during task)	Memory reconsolidation (reactivation + updating) and visuospatial interference (dual-task, mental rotation)	Within 6 hours (74%) or within 72 hours (26%) after trauma	1 guided session (in ED) + optional self-administered booster sessions at home	~20 minutes total (10 min minimum uninterrupted, ~20 min total)	In-person, in the ED waiting area, or bed	Guided by trained research assistants (Psychology MSc students, not clinicians), self-administered thereafter	Participant’s own smartphone with the official Tetris app (Android/iOS)	20/22 (91%) completed the intervention session (2 did not complete for unknown reasons)	Standard ED medical care; no restrictions	Active control (podcast)	Listen to the Swedish Radio philosophy podcast “Filosofiska Rummet” for ~20 minutes on your own smartphone using the SR app, following the instructions to focus on the content.
Freedman et al., 2020 (37)	SnowWorld (semi-immersive virtual reality visuospatial task)	Single-session, acute prevention (memory consolidation disruption)	No (assumed ED context was sufficient memory reminder; no explicit hotspot retrieval)	Visuospatial interference (working memory taxation) – disrupts consolidation of non-verbal trauma memories	Within ~1 hour (range up to 8 hours) after trauma	1 session	Mean 8.06 min (SD 2.02); up to 10 min	In-person, in a small treatment room within the ED	Guided by a research worker (set up VR, instructed to throw snowballs)	Desktop Asus computer, Apple 30-inch Cinema Display screen, wireless mouse, headphones	36/36 (100%) completed the intervention in ED	Standard ED medical care (opioids, stitches, orthopedic assessment noted). No restrictions	No intervention control	No task; completed questionnaires, then waited 10 minutes. Received usual medical care only.
Iyadurai et al., 2018 (38)	Tetris-based cognitive task intervention	Single-session, acute prevention (memory consolidation disruption)	Yes (participants briefly described the worst moments of the accident to the researcher)	Memory consolidation disruption via visuospatial interference (working memory taxation)	Within 6 hours of MVA	1 session	~20 min (minimum 10 min uninterrupted)	In-person, in an ED cubicle	Guided by a clinical psychologist (LI) – not self-administered	Handheld gaming device (Nintendo DS XL) with Tetris game	36/37 (97%) played ≥10 min (1 interrupted by staff)	Usual ED medical care	Attention-placebo control (written activity log)	Pen-and-paper activity log: list activities already done in ED with duration, for ~20 min; no memory reminder cue.
Horsch et al., 2017 (39)	Visuospatial cognitive task (Tetris)	Single-session, acute prevention (memory consolidation disruption)	No (implicit memory reminder via hospital context; no explicit hotspot retrieval)	Visuospatial interference (working memory taxation) – disrupts consolidation of visual trauma memory	Within 6 hours after ECS	1 session	15 minutes (minimum 10 minutes)	In-person, whilst in a hospital bed	Guided by a researcher (not a clinician), participants played on the provided device	Handheld gaming device (Nintendo DS) with Tetris game	26/29 (89.7%) played ≥10 minutes (3 interrupted by medical care)	Usual care (routine obstetric and postnatal care)	Usual care (no intervention)	No task; continued routine clinical care only
Holmes et al., 2017 (40)	Tetris-based cognitive task intervention	Single-session, feasibility (targeting intrusive memories)	Yes (participant asked to think back briefly to traumatic event, specifically worst moments that pop to mind)	Memory reconsolidation (reactivation) + visuospatial interference (Tetris gameplay)	Variable (post-trauma, likely months to years)	1 session	15–20 min of Tetris gameplay (mean 18 min, SD 2.4)	In-person (individual session) with smartphone-based gameplay	Guided by a researcher (verbal instructions); self-administered gameplay on the participant’s own phone	Participant’s own smartphone with downloaded Tetris app (free version)	22/22 (100%) completed ≥15 min of gameplay	None (no other psychological treatment reported)	NA	No control condition

Most diary-based parallel-group RCTs showed reductions in intrusive-memory frequency favouring ICTI, whereas Freedman (37) and Kehyayan (20) did not show a significant benefit (**Table 3**).

**Table 3:** Primary outcome: intrusive memory frequency.

First author, year	Intrusive memory frequency (diary-based)	Intrusion assessment period	Mean/median intrusions – intervention	Mean intrusions – control	Change from baseline (if available)	Number achieving the reduction threshold
Beckenstrom et al., 2026 (21)	Daily diary count of intrusive memories (yes/no + number)	Week 4 (days 22–28), controlling for baseline week	ICTI: median 0.5 (IQR 0.0–5.0)	Active control: median 5.0 (IQR 3.0–11.5); TAU: median 5.0 (IQR 2.5–8.0)	ICTI 72% fewer vs active control; 70% fewer vs TAU	By week 24: 70% of ICTI participants had zero intrusive memories; active control 13%, TAU 20%
Kanstrup et al., 2024 (19)	Daily diary (4x/day for 7 days) – count of intrusive memories of work-related trauma	Week 5 post-intervention (primary endpoint)	Median = 1.0 (IQR 0–3); Mean = 3.4 (SD 8.6)	Median = 5.0 (IQR 1–17); Mean = 12.4 (SD 17.5)	85.9% reduction in mean from baseline (intervention arm: from mean ~21.8 to 3.4). IRR = 0.30 (95% CI 0.17–0.53), p<0.0001	50% of intervention participants had zero intrusive memories at week 5 (complete diary data)
Kehyayan et al., 2024 (20)	Daily diary (once per 24h) – number of intrusions recorded separately for each of the three targeted memories	8 weeks total; pre-intervention (2 weeks) vs post-intervention (6 weeks across two crossover periods)	Tetris: mean pre 2.02 (SD 1.92) → post 1.92 (SD 1.88) (-5.0% change)	Wiki: pre 2.12 (1.89) → post 2.02 (2.18) (-4.7%); No intervention: pre 2.14 (2.22) → post 1.89 (2.19) (-11.7%)	Relative risk (Tetris/Wiki) = 0.947 (95% CI 0.85–1.05), p=0.31; No intervention vs Tetris RR = 1.060 (0.98–1.15), p=0.15; No intervention vs Wiki RR = 1.004 (0.93–1.09), p=0.92	NR
Hardarson et al., 2024 (33)	Daily diary (4 timepoints/day) – total count of intrusive memories	Week 5 (after second intervention session)	Mean 6.98 (SD 5.73), n=8	Baseline (Week -1): mean 25.98 (SD 29.39), n=11	68% reduction from baseline to Week 5 (per protocol)	NR; at 3 months, 91% reduction from baseline
Deforges et al., 2023 (16)	Daily diary of childbirth-related intrusive memories (first week postpartum)	First 7 days postpartum	Per-arm Week 1 diary means and SDs not reported in publication and analysed via Group × Day GLMM only.	Per-arm Week 1 diary means and SDs not reported in publication and analysed via Group × Day GLMM only.	Per-arm Week 1 diary means and SDs not reported in publication and analyzed via Group × Day GLMM only.	Per-arm Week 1 diary means and SDs not reported in publication and analyzed via Group × Day GLMM only.
Iyadurai et al., 2023 (17)	Daily online diary (once per day) – number of intrusive memories of work-related trauma	Week 4 (days 22–28)	Immediate arm: median 1 (IQR 0–3); mean 4.03 (SD 10.68)	Delayed arm: median 10 (IQR 6–17); mean 12.46 (SD 9.28)	IRR = 0.31 (95% CI 0.20–0.48), p<0.001 (immediate vs delayed at week 4)	NR
Deforges et al., 2022 (34)	Daily diary (14 days) of childbirth-related intrusive memories (CB-IMs) – participants ticked a box per day and briefly described the content	Diary 2: first 14 days post-intervention (primary outcome); Diary 3: days 28-42 (weeks 5-6)	Median CB-IMs in diary 2 = 2 (IQR 4); in diary 3 = 2 (IQR 3)	Baseline (diary 1, 14 days pre-intervention): median = 11 (IQR 6)	Median reduction 81.8% (IQR 39.6%) from diary 1 to diary 2; 76.9% (IQR 29.0%) from diary 1 to diary 3	15/17 (88%) had ≥50% reduction from diary 1 to diary 2; 16/17 (94%) from diary 1 to diary 3
Badawi et al., 2021 (35)	Phone app diary (ExpiWell) three times daily – number of intrusive memories	Days 0–21 (baseline period before first Tetris session, then after each session)	NR	NA	Small effect sizes for some participants (e.g., Participant 15: Tau = -0.52, p<0.001 from baseline to after the first session)	NR
Kanstrup et al., 2021 (36)	Pen-and-paper daily diary (4 timepoints per day) for 7 days	Week 1 (days 1–7 post-intervention) (primary timepoint)	Mean 3.85 (SD 8.57), n=20	Mean 7.37 (SD 7.88), n=19	NA	NR
Kanstrup et al., 2021 (week 5) (36)	Same diary method	Week 5 (days 28–34 post-intervention) (secondary timepoint)	Mean 0.28 (SD 0.57), n=18	Mean 2.89 (SD 6.43), n=18	The intervention group had 90% fewer intrusions than the control group at week 5	NR
Freedman et al., 2020 (37)	No daily diary. PTSD Symptom Scale (PSS) – retrospective report of intrusion symptom severity (not frequency count)	2 weeks and 6 months post-trauma	PSS intrusions at 2 weeks: SnowWorld mean 3.54 (SD 3.62), n=28; at 6 months: mean 2.50 (SD 3.26), n=28	PSS intrusions at 2 weeks: Control mean 2.70 (SD 3.45), n=27; at 6 months: mean 2.05 (SD 2.89), n=21	NA	NR
Iyadurai et al., 2018 (38)	Daily pen-and-paper diary (tick box per day/time period) of intrusive memories of the MVA	First 7 days post-accident (day of accident to day 7)	Mean 8.73 (SD 11.55), n=37 (ITT imputed)	Mean 23.26 (SD 32.99), n=34 (ITT imputed)	NA	NR
Horsch et al., 2017 (39)	Daily diary (tick box per day) of intrusive traumatic memories (“flashbacks”) related to ECS	First 7 days post-ECS (day of birth to day 7)	ITT: mean 4.77 (SD 10.71); Per protocol: mean 3.54 (SD 11.16)	ITT: mean 9.22 (SD 10.69); Per protocol: mean 9.00 (SD 9.32)	NA	NR
Holmes et al., 2017 (40)	Daily pen-and-paper diary (ticks per morning/afternoon/evening) – count of intrusive memories of trauma	1 week after the intervention session (post-intervention only; no pre-intervention diary baseline)	Mean 12.65 per week (SD 9.71); ~1.81 per day (n=17)	NA	Not assessed	NR

*In Freedman et al., (37): Trial’s primary outcome was PSS total at 2 weeks (not the intrusion subscale alone). Intrusion subscale shown for comparability with other studies; flagged as different metric from daily diary counts in other trials.

*In Iyadurai et al., (38): Raw means shown; trial’s published d=0.67 was derived from log-transformed analysis to address count data skew.

- Beckenstrom (21): ICTI median 0.5/week vs. active control 5.0 and TAU 5.0.
- Kanstrup (19): ICTI median 1.0 vs. control 5.0; IRR = 0.30.
- Iyadurai (38): ICTI mean 8.73 vs. control 23.26 (62% reduction).
- Horsch (39): ICTI mean 4.77 vs. control 9.22 (48% reduction).
- Hardarson ( (33) – feasibility): 68% reduction at week 5 and 91% at 3 months.
- Kehyayan ( (20) – cross-over): No significant difference (RR = 0.95), possibly due to carry-over effects.

As presented in **Table 4**, most RCTs reported significant reductions in PTSD symptoms favouring ICTI. For example, Beckenstrom (21): PCL-5 effect sizes (Bayes factors) are extremely strong (BF > 1000) vs. controls. Kanstrup (19): PCL-5 OR = 0.21 at 1 month. Iyadurai (17): mean difference -2.29, p < 0.001. Depression and anxiety effects were less consistent; some trials (e.g., Iyadurai (17)) found no significant difference at 4 weeks on PHQ-2, whilst others showed a significant reduction at week 4 but not later. Functioning and quality of life: positive effects were observed, including improved work engagement and reduced burnout (SWEBO), better WHODAS scores, lower insomnia (SCI-08), and higher EQ-5D-5L quality of life. No serious adverse events were attributed to the ICTI. Attrition was generally low and comparable between arms.

**Table 4:** Secondary outcomes: PTSD, depression, anxiety, and functioning.

First author, year	PTSD symptom severity (CAPS, PCL-5, IES-R)	Depression symptoms (PHQ-9, BDI)	Anxiety symptoms (GAD-7, HADS)	Functional outcomes (Work/social functioning)	Sleep outcomes	Distress associated with intrusions	Quality of life	Acute stress symptoms	Emotional impact of memories	Study conclusion
Beckenstrom et al., 2026 (21)	PCL-5: ICTI reduced more than active control (week 4 BF = 1546) and TAU (week 4 BF = 7732). No ICTI participant met probable PTSD criteria by week 12/24.	ICTI reduced at week 4 vs active control (BF = 80.1) and vs TAU (BF = 77.6). Mixed at later timepoints.	ICTI reduced at week 4 vs active control (BF = 13.4) and vs TAU (BF = 53.4). Variable at week 12/24.	WHODAS: extreme evidence for ICTI vs TAU at all timepoints (BFs 508–2230); vs active control BF = 138.1 (week 4), 2.8 (week 12), 21.0 (week 24).	Insomnia reduced for ICTI vs active control (BF = 4.3) and vs TAU (BF = 23.4) at week 4; sustained.	ICTI participants rated less distress/interference vs both comparators, especially at week 24.	EQ-5D-5L: ICTI benefit at week 4 for anxiety/depression vs both comparators; other dimensions null/mixed.	NR	Covered by distress/interference ratings.	EQ-5D-5L: ICTI benefit at week 4 for anxiety/depression dimension (Bayes Factor extreme). Other dimensions null/mixed. At week 12 and 24, evidence weakened. Full BF values available in the original publication.
Kanstrup et al., 2024 (19)	PCL-5 (8-item) lower in intervention at all timepoints (week 1 to 6 months): e.g., week 5 OR = 0.21 (0.10–0.42). IES-R intrusions are lower at all timepoints (OR 0.16–0.25).	NR	NR	WHODAS: better cognition and personal care at 6 months. Lower burnout (SWEBO) at 6 months (OR = 0.46). Less work impact from intrusions (OR 0.22–0.56).	The Sleep Condition Indicator (SCI-02) was better at week 1, 1 month, and 3 months (OR 2.16–3.92), but not at 6 months.	IES-R intrusions (see PTSD). Also, less disruption of concentration due to intrusions (OR ~0.40).	Self-Rated Health (SRHR) was higher (better) in the intervention at all timepoints (OR 2.61–3.13).	NR	Less impact of intrusive memories on daily functioning (OR 0.23–0.47) and occupational functioning (OR 0.22–0.56) across 1–6 months.	A brief, single-session remote ICTI (Tetris + mental rotation) reduces intrusive memories and PTSD symptoms in traumatized healthcare workers, with effects lasting up to 6 months; it is safe, scalable, and superior to active control.
Kehyayan et al., 2024 (20)	NR	NR	NR	NR	NR	NR	NR	NA	NR	In a crossover RCT of PTSD patients (~13.8 years post-trauma), neither Tetris nor verbal control reduced intrusions vs no intervention; possible carryover effects suggest the need for parallel-arm RCTs.
Hardarson et al., 2024 (33)	PCL-5: baseline 36.42 → 3-month 18.71 (d=-0.71). UMT frequency: baseline 3.25 → 3-month 1.29 (d=-2.36). UMT distress: baseline 45.42 → 3-month 24.00 (d=-0.76).	PHQ-9: baseline 11.83 → 3-month 8.29 (d=-0.46)	GAD-7: baseline 8.75 → 3-month 5.00 (d=-0.44)	SDS (Sheehan) functional impairment decreased; WHODAS 2.0 improved; impact of intrusions on daily functioning reduced	Impact on sleep: reduced at 1 month, slight increase at 3 months, but still below baseline; nightmares reduced to near zero at 3 months	UMT distress (see PTSD); intrusion-related distress reduced over time	Not assessed	NA	Vividness and distress ratings reduced; concentration disruption duration decreased.	Remote ICTI in long-standing trauma was feasible and acceptable, with large reductions in intrusive memories (68% at 5 weeks, 91% at 3 months) and improvements in PTSD, depression, anxiety, and functioning; a full RCT is needed.
Deforges et al., 2023 (16)	PCL-5 (self-report): At 6 weeks: marginal effects (total p<0.06; intrusions p<0.0525; arousal p<0.0552). At 6 months: significant total (β=-0.65, p=0.041), alterations in cognition/mood (p=0.0016), and arousal (p<0.0289). CAPS-5 (clinician): No significant group differences at 6 weeks or 6 months.	NR (measured but between-group results NR in publication)	NR (measured but between-group results NR in publication)	NR	NR	NR	NR	NR	NR	A single-session Tetris-based intervention after unplanned cesarean section reduced self-reported CB-PTSD symptoms at 6 months, but not clinician-rated PTSD, and was feasible with preventive potential.
Iyadurai et al., 2023 (17)	PCL-5 (4-item): lower in immediate vs delayed at week 4 (mean diff -2.29, p<0.001). IES-R total lower (diff -0.8, p<0.001).	PHQ-2: no significant difference at week 4 (diff -0.52, p=0.19); but within-arm at week 8 showed a reduction.	GAD-2: lower in immediate at week 4 (diff -0.93, p=0.016).	Work engagement (SWEBO) was higher (diff 0.46, p<0.001); burnout was lower (diff -0.61, p<0.001). Sick days: no difference at week 4 (IRR 1.50, ns); at week 8, within-arm reduction.	Insomnia (SCI-08): better in immediate at week 4 (diff 5.38, p<0.001).	Distress, concentration disruption, and interference all decreased immediately (all p<0.001).	EQ-5D-5L quality of life was higher in immediate (diff 12.32, p=0.002). WHODAS is functioning better (diff = -9.78, p < 0.001).	NA	Lower impact on work functioning and other life areas (both p<0.001).	A brief remote ICTI (Tetris + mental rotation) reduced intrusive memories at 4 weeks in ICU staff, with improvements in PTSD, anxiety, sleep, functioning, and quality of life; it was safe, acceptable, and effects were replicated after crossover, supporting further trials.
Deforges et al., 2022 (34)	PCL-5 (CB-PTSD) total severity: pre 28.71 (SD 12) → post 12.29 (SD 8.84), p<0.001, Hedges’ g=1.47 (large). All 4 symptom clusters significantly reduced (p<0.01 to <0.001, all g>0.8). Of 8 participants with probable CB-PTSD pre-intervention, 0 met criteria post-intervention (p=0.013).	EPDS (Edinburgh Postnatal Depression Scale) pre-intervention: mean 10.67 (SD 4.63) – above clinical cutoff (10.5). Not reported as outcome post-intervention	Not assessed	Not assessed	Not assessed	NR	Not assessed	NA	Participants reported reduced impact on daily life (e.g., less fear of becoming pregnant again) in qualitative feedback.	This is the first test of a single-session behavioral intervention for an old, real-life single-event trauma. Its promising findings support further randomized controlled trials and suggest a potential new approach for CB-PTSD treatment.
Badawi et al., 2021 (35)	PCL-5 total: pre 59.0 (SD 8.0) → post 50.89 (17.51), Hedge’s g=0.47 (n=9). IES-R intrusions: pre 30.56 (5.36) → post 28.00 (6.38), g=0.37	HADS depression: pre 12.25 (4.86) → post 10.67 (4.03), g=0.38 (n=8)	HADS anxiety: pre 15.00 (2.65) → post 13.11 (4.60), g=0.38 (n=9)	Not assessed	Not assessed	Not assessed	Not assessed	NA	Not assessed	A feasibility study showed that Tetris as an adjunct to trauma-focused therapy is feasible in inpatient PTSD care, with small to moderate reductions in intrusions and modest improvements in PTSD, depression, and anxiety; optimal dosing requires further study.
Kanstrup et al., 2021 (36)	IES-R total at 1 week: Intervention mean 21.3 (SD 15.4), Control 28.0 (16.9), d=0.41 (-0.26,1.07). At 1 month: Intervention 15.0 (12.6), Control 22.5 (15.2), d=0.52. At 6 months: Intervention 19.5 (21.1), Control 16.9 (16.5), d=-0.15	HADS depression subscale at 1 week: Intervention 1.43 (1.91), Control 2.17 (2.50), d=0.33 (-0.41,1.06)	HADS anxiety subscale at 1 week: Intervention 2.14 (2.28), Control 3.11 (2.70), d=0.38 (-0.35,1.12)	Work and Social Adjustment Scale (WSAS) at 1 week: Intervention 10.0 (11.4), Control 14.9 (11.0), d=0.45 (-0.23,1.12)	Self-rated sleep (SRSR) at 1 week: Intervention 5.91 (2.35), Control 5.00 (2.98), d=-0.38 (-1.11,0.36)	IES-R intrusion subscale at 1 week: Intervention 1.15 (0.94), Control 1.60 (0.88), d=0.49 (-0.19,1.17)	Self-rated health (SRHR) at 1 week: Intervention 5.82 (1.10), Control 5.37 (1.21), d=-0.40 (-1.12,0.33)	NA	Concentration disruption due to intrusions at 1 week: Intervention 2.30 (2.64), Control 2.45 (2.52), d=0.06 (-0.67,0.79)	A brief single-session ICTI (Tetris + mental rotation) delivered acutely in the Emergency Department reduced intrusive memories at 1 week in trauma-exposed adults, with the intervention group showing 90% fewer intrusions than control at Week 5. The pilot trial supports the feasibility and exploratory efficacy of ICTI for acute trauma in an emergency care setting.
Freedman et al., 2020 (37)	PSS total score at 2 weeks: SnowWorld 10.39 (11.62), Control 7.52 (9.69), p=0.22; probable PTSD: 28.6% vs 22.2%, ns. At 6 months: SnowWorld 7.93 (10.11), Control 5.90 (6.64), p=0.64; probable PTSD: 21.4% vs 14.3%, ns.	Not assessed	Not assessed	Not assessed	Not assessed	Not assessed	Not assessed	Not assessed	Not assessed	This pilot RCT found no benefit of a single VR visuospatial task (SnowWorld) vs no intervention on PTSD symptoms at 2 weeks or 6 months; it was feasible but did not replicate prior Tetris findings, possibly due to methodological differences.
Iyadurai et al., 2018 (38)	IES-R intrusion subscale at 1 week: Intervention 7.27 (5.27), Control 10.70 (7.29), p=0.024, d=0.54. PDS total at 1 week: ns (d=0.20). At 1 month: No significant differences on IES-R or PDS. PTSD diagnosis at 1 month (PDS): Intervention 12.9%, Control 9.7%, OR = 1.40, ns.	HADS total at 1 week and 1 month: no significant differences (d=0.15 and 0.04)	HADS total (combined anxiety/depression) – no differences	Not assessed	Not assessed	IES-R intrusion subscale (see PTSD) – significant at 1 week only	Not assessed	Not assessed	Not assessed	A single Tetris session with a memory cue within 6 hours post-MVA reduced intrusive memories at 1 week (62% vs active control), with effects limited to intrusion symptoms; it was feasible, acceptable, and safe, with no 1-month benefit.
Horsch et al., 2017 (39)	PDS (Posttraumatic Diagnostic Scale) at 1 month: total score ITT: Intervention 13.5 vs Control 6.8 (p=ns). Per protocol: no significant differences. PTSD diagnosis at 1 month: ITT 12.1% vs 25.1% (ns)	HADS depression at 1 week and 1 month: no significant group differences (ITT and per protocol)	HADS anxiety at 1 week and 1 month: no significant group differences	Not assessed	Not assessed	Not assessed	Not assessed	ASDS total score at 1 week: no significant difference. ASDS avoidance, arousal: no differences	Not assessed	A single 15-min Tetris session within 6 hours post-cesarean reduced intrusive memories (~48%) in the first week vs usual care, with good feasibility and safety; no significant PTSD difference at 1 month.
Holmes et al., 2017 (40)	Not assessed	Not assessed	Not assessed	Concentration disruption: 82% reported >1 min disruption per intrusion; 68% >5 min; 32% >30 min. General concentration difficulties (0-10): mean 5.68, SD 3.59. Positive correlation with intrusion frequency (r=0.64, p=0.001).	Not assessed	Not assessed	Not assessed	NA	Not assessed	A feasibility study among young refugees (n=22) showed that diary monitoring, combined with a single Tetris session after a memory cue, was feasible, acceptable, and well-received, supporting the further development of scalable smartphone-based interventions.

### Depression outcomes

Depression was assessed in four studies using different measures: HADS-D [Iyadurai (38), Kanstrup (36), Horsch (39)], PHQ-2 [Beckenstrom (21), and Iyadurai (17)]. No meta-analysis was performed due to the heterogeneity of instruments and the insufficient number of studies reporting comparable metrics. Individually, Beckenstrom (21) reported strong Bayesian evidence for ICTI reducing PHQ-2 scores at week 4 compared to active control (BF = 80.1) and TAU (BF = 77.6), though effects were mixed at later timepoints. Iyadurai (17) found no significant difference in PHQ-2 at week 4 (mean difference –0.52, 95% CI –1.23 to 0.19). Kanstrup (36) reported a small effect favouring ICTI at 1 week (Cohen’s d = 0.33, 95% CI –0.41 to 1.06). Horsch (39) found no significant group differences at 1 week or 1 month. Overall, evidence for an effect of ICTI on depression is inconsistent and limited by small numbers and heterogeneous measures.

### Anxiety outcomes

Anxiety was assessed using GAD-2 [Beckenstrom (21), Iyadurai (17)], and HADS-A [Kanstrup (36), Horsch (39)]. Meta-analysis was not performed due to the heterogeneity of measures and limited comparable data. Beckenstrom (21) reported strong evidence for ICTI reducing GAD-2 at week 4 versus active control (BF = 13.4) and TAU (BF = 53.4), though later timepoints were mixed. Iyadurai (17) found a significant difference in GAD-2 favouring immediate ICTI at week 4 (mean difference –0.93, 95% CI –1.68 to –0.17). Kanstrup (36) reported a small effect at 1 week (Cohen’s d = 0.38, 95% CI –0.35 to 1.12). Horsch (39) found no significant differences at 1 week or 1 month. Collectively, the evidence tentatively favours ICTI for reducing anxiety in the short term, but larger controlled trials with consistent measures are needed.

### Between-group mean difference in intrusive memory frequency (ICTI vs. Control) at primary timepoint

A random-effects meta-analysis of raw mean differences (ICTI minus control) was conducted for the five parallel-group RCTs that reported intrusive memory frequency per week. The pooled mean difference was –6.98 intrusive memories per week (95% CI –9.84 to –4.13), favouring the ICTI intervention. The effect was statistically significant as the confidence interval did not cross zero. Heterogeneity was low (I^2^ = 21.9%, p = 0.275), indicating that the five studies provided a consistent estimate of the treatment effect. Individual study estimates ranged from –14.53 to –3.52, with three of the five studies showing statistically significant benefits favouring ICTI. The forest plot is presented in **Figure 2**. Randomized studies that could not be included in the primary raw mean-difference meta-analysis because of incompatible design or outcome format are summarized in the qualitative and narrative synthesis below.

**Figure 2:**
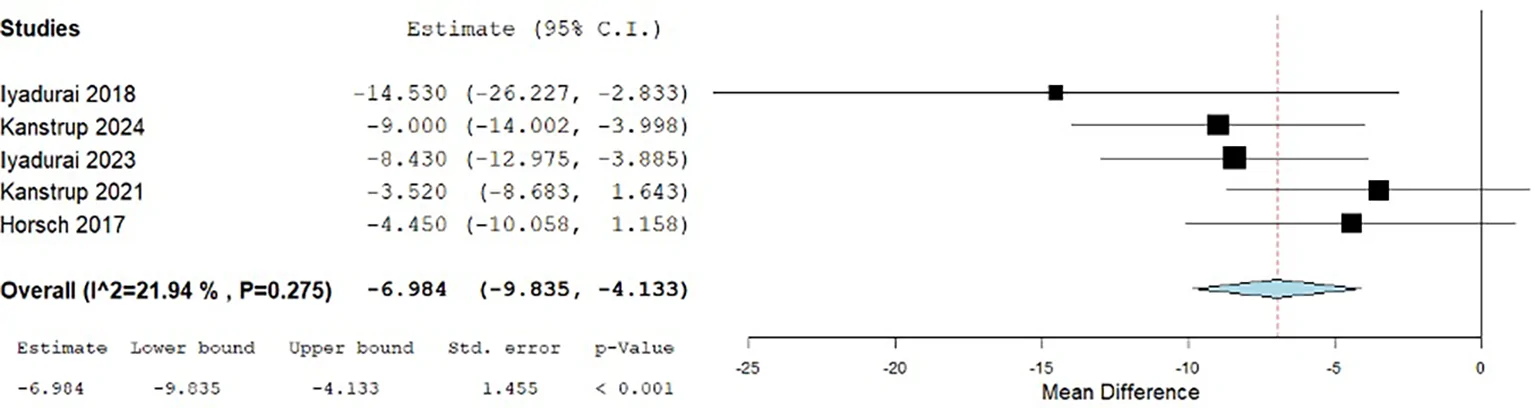
Forest plot of between-group raw mean differences in intrusive memory frequency per week. (Parallel-Group RCTs Only).

### Moderator analysis – memory reactivation cue

A random-effects subgroup analysis was conducted to examine whether the presence of an explicit memory reactivation (MR) cue modified the intervention effect on intrusive memory frequency **Figure 3**. Amongst the four studies that used an explicit MR cue (Iyadurai (38), Kanstrup (19), Iyadurai (17), and Kanstrup (36)), the pooled mean difference was −7.65 intrusive memories per week (95% CI −11.01 to −4.29), favouring ICTI, with low heterogeneity (I^2^ = 28.1%, p = 0.243). The single study without an explicit MR cue (Horsch (39)) showed a mean difference of −4.45 intrusive memories per week (95% CI −10.06 to 1.16). Although the confidence intervals overlapped, suggesting no clear difference in treatment effect between cue and no-cue studies, this comparison should be interpreted with considerable caution. Because the no-cue subgroup included only one study, a reliable formal test for subgroup differences could not be estimated. Therefore, this analysis should be considered exploratory and hypothesis-generating rather than definitive.

**Figure 3:**
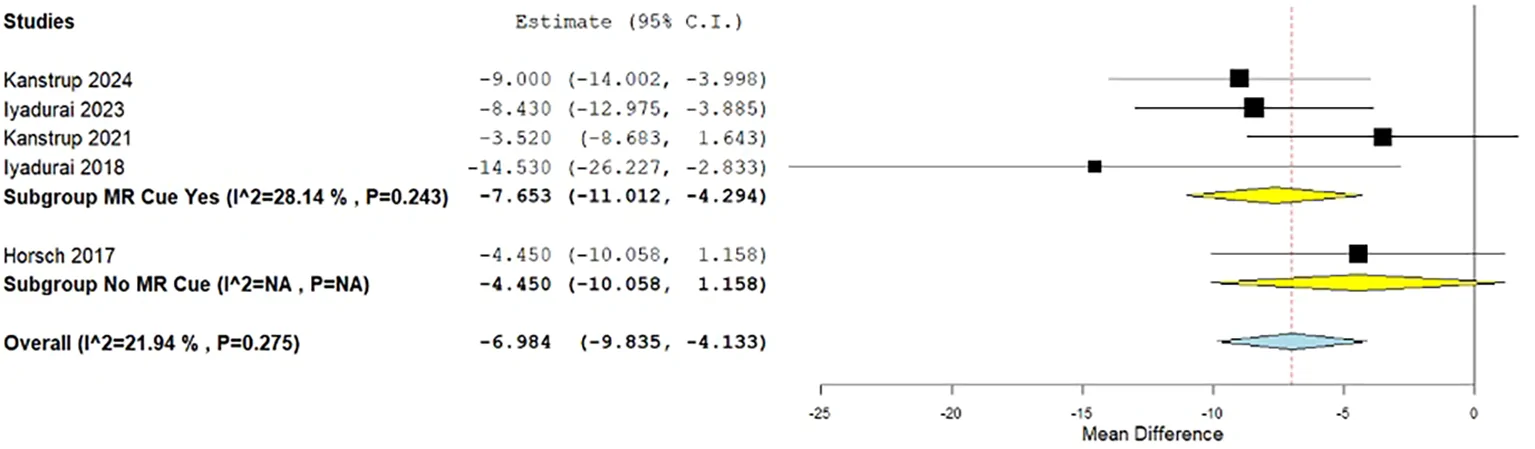
Forest plot of between-group mean difference in intrusive-memory frequency according to the presence of an explicit memory reactivation cue.

### PTSD symptom severity (1–4 weeks)

The forest plot suggests that the intervention was associated with lower PTSD symptom severity compared with control, with an overall pooled mean difference of -2.42 (95% CI -4.40 to -0.44) **Figure 4**. Because the confidence interval does not cross zero, this overall effect is statistically significant. However, the results were not fully consistent across studies, as shown by moderate-to-substantial heterogeneity (I^2^ = 60.88%, p = 0.037), indicating that the study estimates varied meaningfully. The clearest benefit appears in the 1-month subgroup, where the pooled effect was also significant (MD = -2.98, 95% CI -4.74 to -1.23; I^2^ = 52.69%), whilst the less-than-1-month subgroup was based on a single study and showed a positive but imprecise estimate (MD = 2.87, 95% CI -2.78 to 8.52), so no firm conclusion can be drawn from it. Overall, the pattern favours the intervention, but the moderate heterogeneity suggests that the magnitude of benefit may differ across studies and follow-up timepoints.

**Figure 4:**
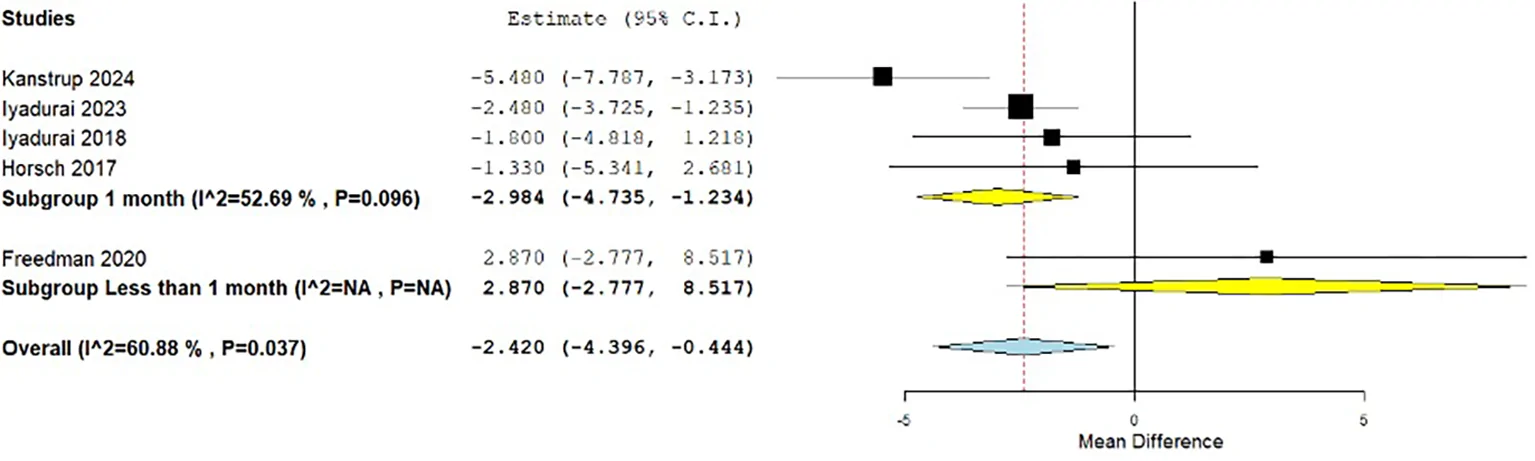
Forest plot of PTSD symptom severity (1–4 weeks post-intervention).

It is worth noting that different PTSD instruments (PCL-5, IES-R, PDS/ASDS, PSS) were pooled as raw mean differences. This limits clinical interpretability, as the scales have different ranges; results should be interpreted with caution.

### Crossover trial sensitivity analysis

A standalone sensitivity analysis of the crossover study by Kehyayan et al. (20) was conducted to evaluate the intervention effect within this within-subject design, yielding a pooled risk ratio of 0.95 (95% CI 0.85 to 1.05), which was not statistically significant (Z = 1.00, p = 0.32) **Figure 5**. This estimate indicates no clear benefit of the visuospatial intervention over the verbal control condition in this single crossover trial, as the confidence interval crosses the null value of 1.0. This null finding contrasts with the significant pooled effect observed in the five parallel-group RCTs, and may be attributable to methodological factors inherent to the crossover design, such as potential carryover effects in the absence of a washout period, or to the chronic, long-standing nature of the trauma in this population (mean 13.8 years post-trauma), which may be less responsive to a single-session intervention. Overall, this sensitivity analysis reinforces the decision to restrict the primary quantitative synthesis to parallel-group RCTs whilst highlighting the need for further parallel-arm trials in chronic populations.

**Figure 5:**
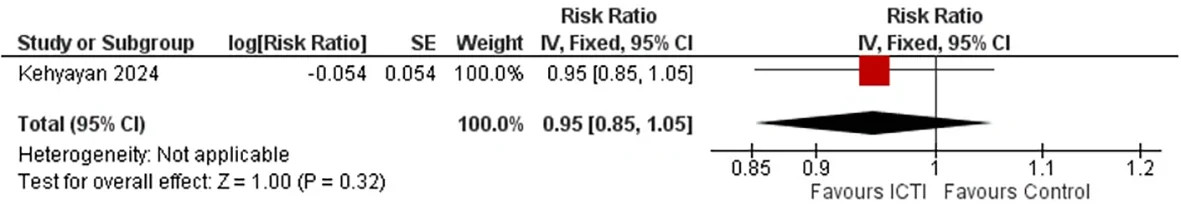
Standalone sensitivity analysis of the crossover trial (risk ratio for intrusive memory frequency).

### Qualitative and narrative synthesis

Seven studies were not included in the primary raw mean-difference meta-analysis of weekly diary-recorded intrusive-memory frequency. These comprised three randomized trials with outcome formats or designs incompatible with raw mean-difference pooling, and four non-randomized feasibility or pre–post studies. These studies were therefore summarized narratively to contextualize the pooled findings and to describe evidence from different populations, intervention formats, and outcome measurement approaches.

#### Randomized trials not included in the primary raw mean-difference meta-analysis

Three randomized trials were not included in the primary pooled raw mean-difference analysis because their data were not directly compatible with the five diary-count RCTs included in the main forest plot.

Beckenstrom et al. (21) conducted a three-arm Bayesian adaptive randomized trial amongst UK NHS healthcare staff exposed to COVID-19-related occupational trauma. The study compared ICTI with an active control condition and treatment as usual. At the Week 4 primary endpoint, the ICTI group had substantially fewer intrusive memories than both comparators, with a median of 0.5 intrusive memories per week compared with 5.0 per week in the active control group and 5.0 per week in the treatment-as-usual group. The Bayesian analysis provided strong evidence favouring ICTI over both active control and treatment as usual. Although this study was not included in the raw mean-difference meta-analysis because of its three-arm Bayesian adaptive design and use of model-based estimates, its findings were directionally consistent with the pooled effect observed in the primary analysis.

Deforges et al. (16) evaluated a single-session Tetris-based intervention after unplanned cesarean section in a multicentre, double-blind randomized trial. The primary outcome was childbirth-related PTSD symptom severity rather than diary-recorded intrusive-memory frequency. At 6 weeks, self-reported PCL-5 total scores and intrusion subscale scores showed marginal effects favouring the intervention, whilst clinician-rated CAPS-5 outcomes did not show significant between-group differences. At 6 months, self-reported PCL-5 total scores favoured the intervention. Although Week 1 diary data were collected, per-arm means and standard deviations were not reported, and the diary outcome was analysed using a group-by-day model. Therefore, this trial could not be included in the primary raw mean-difference meta-analysis. Overall, the trial provides supportive but indirect evidence, with stronger effects on self-reported PTSD symptoms than on clinician-rated outcomes.

Freedman et al. (37) was a pilot randomized trial of SnowWorld, a semi-immersive virtual reality visuospatial task, in emergency department trauma survivors. This study differed from the Tetris-based ICTI trials in both intervention type and outcome measurement. It did not use a daily diary of intrusive-memory frequency; instead, outcomes were assessed using the Posttraumatic Symptom Scale. At 2 weeks, PSS intrusion scores were numerically higher in the SnowWorld group than in the control group, and the trial did not demonstrate a significant benefit on PTSD symptoms at either 2 weeks or 6 months. This study therefore represents an important exception to the generally favourable pattern of findings and suggests that the therapeutic effect observed in Tetris-based ICTI studies may not automatically generalize to all visuospatial tasks or to questionnaire-based PTSD outcomes.

#### Non-randomized studies

Four non-randomized studies were included in the narrative synthesis only because they lacked a concurrent randomized control group or did not provide data suitable for controlled effect-size pooling.

Hardarson et al. (33) evaluated a remotely delivered imagery-competing task intervention in 12 trauma-exposed women with long-standing intrusive memories, with a mean time since trauma of approximately 20.7 years. The intervention was feasible and acceptable, and participants showed large reductions in intrusive memories, including a 68% reduction at Week 5 and a 91% reduction at 3 months. PTSD, depression, anxiety, and functioning also improved over follow-up. However, because the study used a feasibility pre–post design without a concurrent control group, these improvements cannot be attributed definitively to the intervention.

Deforges et al. (34) tested a single-session intervention combining childbirth-related memory reactivation and Tetris gameplay in women with childbirth-related intrusive memories. Participants showed a large median reduction in childbirth-related intrusive memories after the intervention, and most achieved at least a 50% reduction. PTSD symptom severity also improved substantially. This proof-of-principle study supports the feasibility of targeting long-standing childbirth-related intrusive memories, but its uncontrolled design means that the findings should be interpreted as preliminary.

Badawi et al. (35) examined adjunctive Tetris gameplay after imaginal exposure sessions in an inpatient PTSD treatment setting. The study suggested that adding a visuospatial task after trauma-focused therapy was feasible, with small-to-moderate reductions in intrusion-related and PTSD outcomes. However, the study was limited by attrition, small sample size, and the absence of a separate control group, making it difficult to isolate the specific effect of Tetris from the broader inpatient trauma-focused treatment programme.

Holmes et al. (40) assessed feasibility and acceptability of a Tetris-based intervention amongst young refugees aged 16–25 years. The study demonstrated that participants could complete diary monitoring and smartphone-based Tetris gameplay, and intrusive memories were commonly associated with concentration disruption. This study did not test efficacy using a controlled design, but it supports the feasibility of delivering a simple, scalable intervention in a refugee population.

#### Integrated narrative interpretation

Overall, the qualitative and narrative evidence was broadly consistent with the primary meta-analysis, particularly amongst Tetris-based studies using memory reactivation and diary-based intrusive-memory outcomes. Beckenstrom et al. (21) provided strong randomized evidence favouring ICTI despite being incompatible with raw mean-difference pooling, whilst Deforges et al. (16) suggested possible benefits on self-reported childbirth-related PTSD symptoms but provided less direct evidence for diary-recorded intrusive-memory frequency. The non-randomized studies further supported feasibility, acceptability, and potential clinical utility across diverse settings, including remote delivery, childbirth-related trauma, inpatient PTSD treatment, and young refugees.

However, the narrative synthesis also highlights important limitations. Evidence from uncontrolled studies cannot establish causal efficacy, and improvements may reflect regression to the mean, natural recovery, concurrent treatment, or repeated monitoring. Freedman et al. (37) was the clearest exception to the overall favourable pattern, showing no significant benefit of SnowWorld VR compared with control. This may reflect differences in intervention content, absence of an explicit memory reactivation cue, shorter task exposure, or use of questionnaire-based PTSD outcomes rather than prospective diary-recorded intrusive-memory frequency. Taken together, the narrative synthesis supports the plausibility and feasibility of ICTI across populations, whilst reinforcing that the strongest efficacy evidence comes from the five parallel-group diary-based RCTs included in the primary meta-analysis.

### Funnel plot of primary intrusive-memory

**Figure 6** presents the funnel plot of the five studies in the primary meta-analysis. Visual inspection showed no obvious asymmetry, with studies distributed reasonably around the pooled estimate. However, because fewer than 10 studies were available, formal statistical tests are underpowered and were not performed. No definitive conclusion regarding publication bias can be drawn from this small sample.

**Figure 6:**
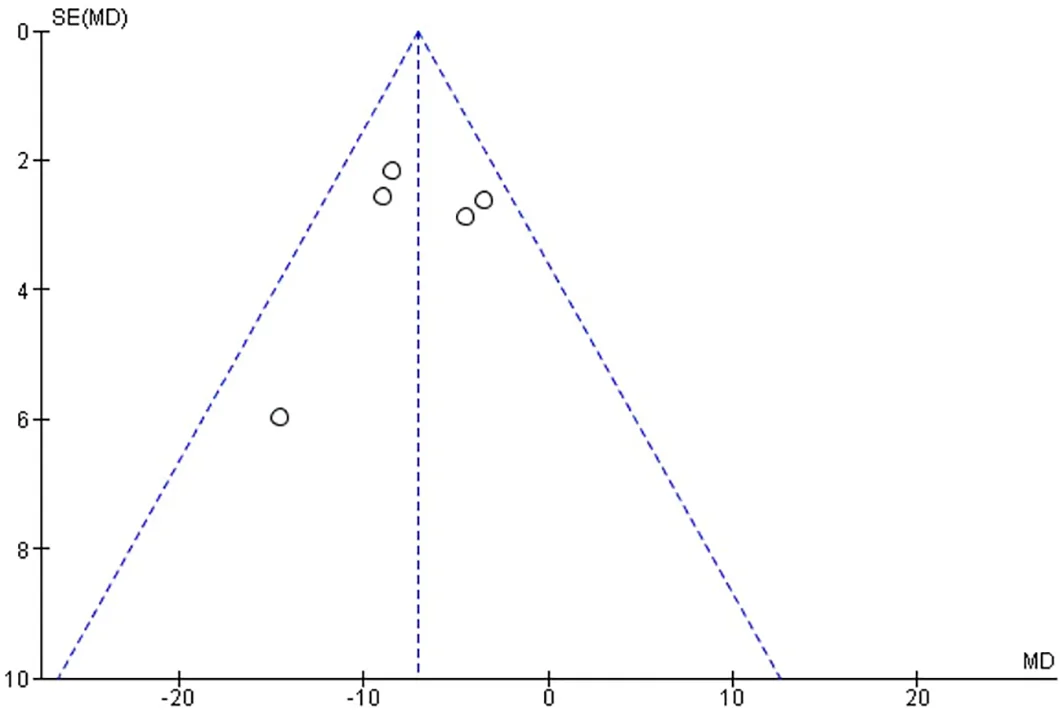
Funnel plot of primary intrusive-memory frequency (n = 5).

### Risk of bias assessment

In **Figure 7**, the ROB 2 assessment indicates that of the 9 included RCTs, 2 were judged at low risk of bias overall, 4 had some concerns, and 3 were at high risk of bias, supporting the internal validity of the evidence base. Several studies, including Kanstrup et al. (19), Deforges et al. (16), and the most recent reports, showed low risk across nearly all domains and were judged as low risk overall (16, 19). In contrast, a few studies raised concerns or were rated as high risk, mainly due to deviations from the intended intervention, missing outcome data, or selective reporting. Notably, Kanstrup et al. (36), Freedman et al. (37), and Horsch et al. (39) were judged as high risk overall, reflecting weaknesses in one or more key domains rather than a uniform lack of quality (36, 37, 39). Therefore, the figure indicates that the evidence is generally reliable, but the findings should still be interpreted with some caution because a minority of studies contributed a higher risk of bias that could have influenced the estimated effects.

**Figure 7:**
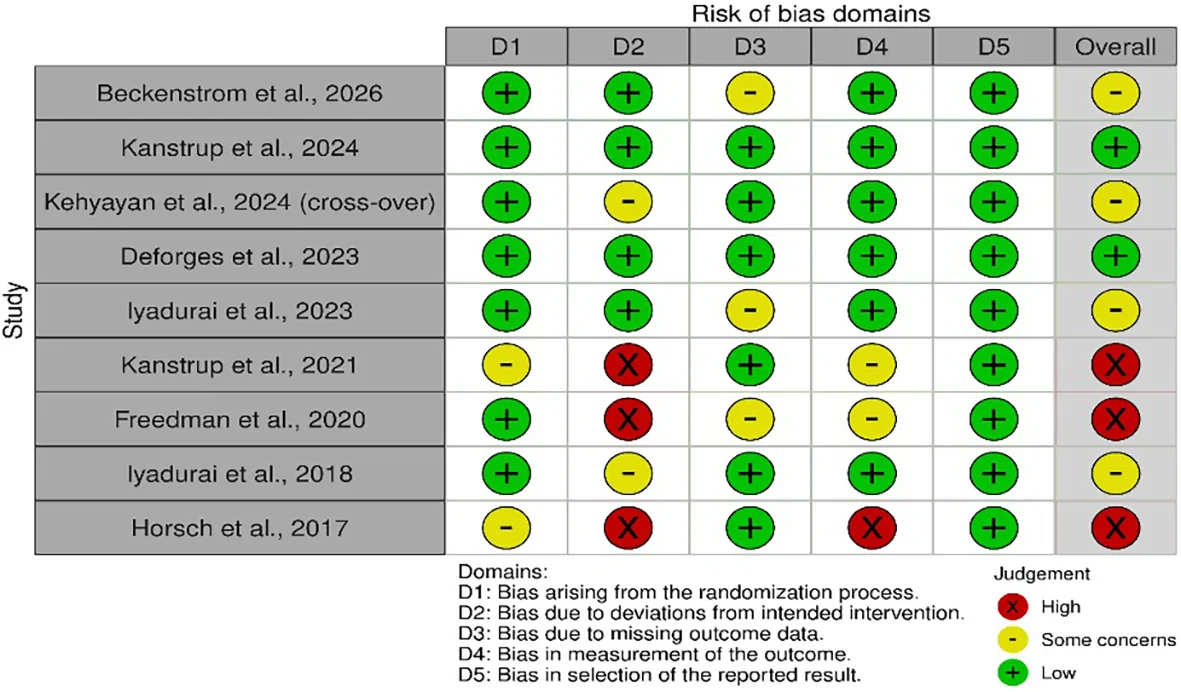
Risk of bias assessment of the included randomized controlled trials (RoB2).

As shown in **Figure 8**, the ROBINS-I assessment indicates that the non-randomized evidence was generally limited by important methodological concerns, with all included studies judged to be at serious overall risk of bias. The main source of bias was confounding, which was rated serious across every study, suggesting that baseline differences and other uncontrolled factors may have influenced the observed effects. In addition, most studies reported moderate concerns about participant selection, deviations from intended interventions, and, in some cases, missing data, although classification of the interventions, outcome measurement, and selection of the reported results were mostly rated as low risk.

**Figure 8:**
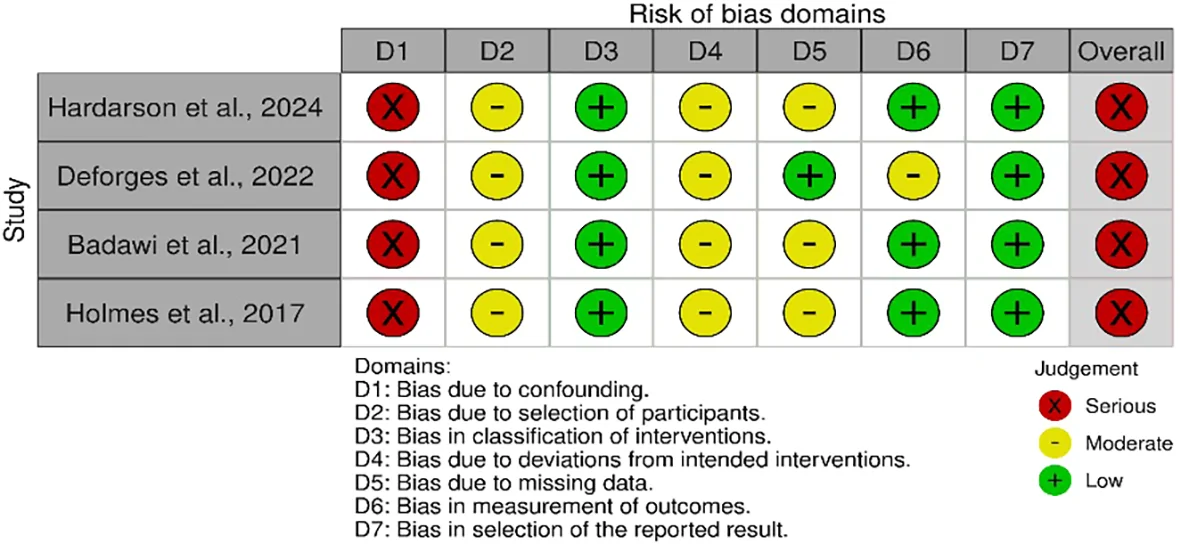
Risk of bias assessment of the non-randomized studies (ROBINS-I).

### GRADE assessment

The GRADE assessment revealed moderate-certainty evidence that the ICTI substantially reduces intrusive memory frequency compared to control conditions (**Table 5**). Across five parallel-group randomized controlled trials, 398 participants were randomized, of whom 359 contributed analyzable primary-outcome data to the pooled raw mean-difference analysis. ICTI reduced intrusive memories by a mean difference of −6.98 intrusions per week (95% CI −9.84 to −4.13), favouring the intervention. Certainty of evidence for this outcome was downgraded by one level for risk of bias, because two of the five pooled trials (Kanstrup (36), Horsch (39)) were judged at high overall risk of bias on RoB 2, and a further two (Iyadurai (38), Iyadurai (17)) had some concerns; only one pooled trial (Kanstrup (19)) was judged low risk. For PTSD symptom severity, the evidence was rated as low certainty due to serious inconsistency (I^2^ = 60.9%, p = 0.037) and indirectness arising from the pooling of different PTSD instruments (PCL-5, IES-R, PDS/ASDS, PSS) as raw mean differences. The pooled mean difference of –2.42 points (95% CI –4.40 to –0.44) suggests a modest reduction in PTSD symptoms favouring ICTI, but this finding should be interpreted with caution given the heterogeneity and measurement differences across studies. Publication bias was rated as undetected for the primary outcome, as a funnel plot of the five studies showed no obvious asymmetry; however, formal statistical testing was not performed due to the small number of studies (k < 10), and definitive conclusions about publication bias cannot be drawn. Overall, the evidence provides moderate certainty that ICTI reduces intrusive memories, reflecting residual concerns about risk of bias in the contributing trials, and the effect on PTSD symptom severity is less certain and requires further investigation with standardised outcome measures in larger trials.

**Table 5:** GRADE summary of findings for imagery-competing task intervention vs. control.

Certainty assessment							№ of patients (n = 359 Analyzed)		Effect		Certainty	Importance
№ of studies	Study design	Risk of bias	Inconsistency	Indirectness	Imprecision	Other considerations	ICTI	Control	Relative (95% CI)	Absolute (95% CI)		
Intrusive memory frequency (follow-up: range 1 weeks to 4 weeks; assessed with: Daily diary)												
5	randomised trials	serious^d^	not serious	not serious	not serious	none^a^	182	177	MD –6.98 (–9.84 to –4.13)	6.98 fewer intrusions per week 95% CI: 9.84 fewer to 4.13 fewer	⨁⨁⨁◯ Moderate^da^	CRITICAL
PTSD symptom severity (follow-up: range 1 week to 4 weeks; assessed with: PCL-5, IES-R, PDS/ASDS, PSS; Scale from: 0 to 80)												
5	randomised trials	not serious	serious^b^	serious^c^	not serious	none	285	286	MD –2.42 (–4.40 to –0.44)	MD 2.42 points on scale lower (4.4 lower to 0.44 lower)	⨁⨁◯◯ Low^b,c^	CRITICAL

CI, confidence interval; MD, mean difference.

*Explanations.*

^a^A funnel plot was generated for visual inspection of the five studies included in the primary meta-analysis. In accordance with the Cochrane Handbook, formal statistical tests (e.g., Egger’s regression test) were not performed because they are underpowered with fewer than 10 studies. The funnel plot showed no obvious asymmetry, but definitive conclusions regarding the presence or absence of publication bias cannot be drawn from this small set of studies. Therefore, publication bias was rated as undetected, with no downgrade applied, but this limitation is acknowledged narratively.

^b^I^2^ = 60.9%, p = 0.037.

^c^Different instruments pooled as raw MD.

^d^Downgraded one level for risk of bias: two of the five pooled trials [Kanstrup (36), Horsch (39)] were judged at high overall risk of bias on RoB 2, and two further trials [Iyadurai (38), Iyadurai (17)] had some concerns; only one trial [Kanstrup (19)] was judged low risk.

## Discussion

This systematic review and meta-analysis evaluated visuospatial imagery-competing task interventions for reducing intrusive memories after real-world trauma. Across five parallel-group randomized controlled trials that reported weekly diary-recorded intrusive-memory frequency, ICTI reduced intrusive memories compared with control conditions. The pooled raw mean difference was −6.98 intrusive memories per week, with a 95% confidence interval from −9.84 to −4.13 and p < 0.001. Heterogeneity was low, with I^2^ = 21.94%, and the certainty of evidence for this primary outcome was rated as moderate, downgraded one level for risk of bias because two of the five pooled trials were judged at high overall risk of bias. The PTSD symptom severity analysis also favoured ICTI, with a pooled mean difference of −2.42 points, but the certainty of evidence was low because different PTSD instruments with different score ranges were combined as raw mean differences and heterogeneity was moderate. Depression and anxiety outcomes could not be pooled because measures and reporting formats differed across studies. The qualitative and narrative synthesis generally aligned with the primary finding, especially in studies that used Tetris-based interventions and prospective diary measures of intrusive-memory frequency. Randomized studies that were not included in the primary meta-analysis also tended to support the beneficial effect.

The present findings extend previous laboratory and translational reviews by focusing on real-world trauma rather than analogue trauma exposure. Asselbergs et al. reported a moderate effect of cognitive interventions on intrusive memories in trauma film experiments, with visuospatial interference tasks showing clearer benefit than verbal tasks (7). Varma et al. later examined a wider range of lab-analogue methods and found that techniques designed to reduce intrusive memories produced small to moderate effects, with trauma reminder plus Tetris showing benefit when administered immediately after, or after a delay following, analogue trauma (6). Astill Wright et al. reviewed consolidation and reconsolidation therapies for PTSD and re-experiencing, and identified cognitive task memory interference with memory reactivation as one of the few protocols with evidence of benefit for intrusive memories (15). Our review agreed with these prior syntheses but moved the evidence base closer to clinical translation by restricting the primary meta-analysis to real trauma populations and diary-recorded outcomes.

The pooled finding also aligned with the symptom focused framework proposed by Iyadurai et al., which treats intrusive memories as a clinically relevant target linking cognitive science and clinical application (41). The real-world trials included in the primary analysis showed a consistent direction of effect across emergency department trauma, emergency caesarean section, and occupational trauma settings (17, 19, 36, 38, 39). Non-pooled randomized evidence gave a more mixed picture. The Bayesian adaptive trial by Beckenstrom et al. supported the same direction of effect, although its model-based reporting and three-arm design were not compatible with raw mean-difference pooling (21). The START trial by Deforges et al. provided supportive but indirect evidence because the main extractable outcome was childbirth-related PTSD symptom severity rather than per-arm diary frequency (16). By contrast, Freedman et al. found no significant benefit of SnowWorld VR, and Kehyayan et al. found no significant benefit of Tetris over verbal control in a crossover trial of chronic PTSD (20, 37). These findings suggest that intervention content, reactivation procedures, trauma chronicity, and outcome measurement may influence observed effects.

Several mechanisms may explain why ICTI reduced diary recorded intrusive memory frequency. Dual representation theory proposes that traumatic memories can be stored with strong sensory-perceptual representations and weaker contextual integration, which may increase involuntary sensory intrusions (42). Cognitive models of PTSD similarly emphasize data-driven trauma encoding, negative appraisals, and poorly integrated autobiographical memory as factors that maintain re-experiencing symptoms (43). Within this framework, a brief reminder may reactivate the trauma image, whilst Tetris or another visuospatial task taxes the same limited visuospatial working memory resources needed to maintain or restabilise vivid imagery.

Experimental work supports this account. James et al. (44) showed that Tetris after memory reactivation reduced intrusive memories of experimental trauma, and early cognitive-science studies proposed that visuospatial gameplay could compete with the build-up of visual flashbacks (4). In the present review, the memory reactivation subgroup analysis was compatible with this interpretation, but it did not prove mechanism because the no-cue subgroup contained only one study. The diary-based primary outcome is also relevant. Daily diary methods capture prospective intrusive-memory counts and avoid some limitations of retrospective questionnaire scoring, although they still depend on participant adherence and interpretation of diary instructions (45). The mechanism should therefore be viewed as plausible rather than established.

Current PTSD guidelines recommend active monitoring or trauma-focused psychological therapy after early clinically important symptoms, and established PTSD treatments such as trauma-focused CBT, cognitive processing therapy, prolonged exposure, and EMDR remain the main recommended interventions (12, 46, 47). ICTI is not yet positioned within these guidelines as a standard treatment or preventive intervention. The present findings do not challenge those recommendations. They support a narrower conclusion: a brief, targeted, low intensity intervention may reduce intrusive-memory frequency after real world trauma and may be suitable for further testing as an adjunct or early symptom-focused strategy.

The next clinical trials should use adequately powered, parallel-group designs in clearly defined trauma populations. Acute trials should recruit emergency department and maternity patients within prespecified time windows and should report weekly diary counts, PTSD diagnosis or severity at one and three months, adverse events, and functional outcomes. Delayed intervention trials should test occupational trauma and chronic intrusive memories using the same diary outcome and a standardized PTSD scale. Factorial designs could test whether memory reactivation is necessary by comparing Tetris with and without an explicit hotspot cue.

This review had several strengths. It focused on real-world trauma rather than laboratory analogue trauma, separated diary-based RCTs from studies with incompatible outcome formats, used a clinically interpretable raw mean difference, and evaluated certainty of evidence. It also distinguished the primary pooled analysis from narrative evidence, which reduced the risk of mixing questionnaire outcomes, Bayesian estimates, crossover data, and uncontrolled pre-post designs in a single effect estimate.

Several limitations remain. The primary meta-analysis included only five RCTs, which limited precision for moderator analyses, funnel plot interpretation, and assessment of publication bias. The PTSD severity analysis was exploratory because different instruments were pooled as raw mean differences. Residual heterogeneity may reflect trauma type, intervention timing, comparator intensity, delivery mode, and follow-up duration. One analytic decision differed from the initial broad plan: the primary pooled analysis was restricted to parallel-group RCTs with diary-count data rather than combining incompatible metrics. This improved interpretability but reduced the number of pooled studies.

## Conclusion

ICTI reduced weekly diary-recorded intrusive-memory frequency in five parallel-group randomized trials of real world trauma. The effect was clinically interpretable and supported by moderate-certainty evidence, downgraded from high for residual risk of bias in the contributing trials. Evidence for broader PTSD symptom severity was low certainty, and evidence for depression, anxiety, and functional outcomes remained less conclusive. ICTI should therefore be considered a promising targeted intervention for intrusive memories rather than an established standalone treatment for PTSD. Larger independent trials with standardized diary and PTSD outcomes are needed before routine clinical implementation.

## Data Availability

The original contributions presented in the study are included in the article/**Supplementary Material**. Further inquiries can be directed to the corresponding author.
