# Supplementary material for: Visuospatial imagery-competing task interventions for reducing intrusive memories following real-world trauma: a systematic review and meta-analysis
Source: Front Psychiatry. 2026 Oct 13;17:1925949. doi: 10.3389/fpsyt.2026.1925949 (PMC13710394; doi:10.3389/fpsyt.2026.1925949)
Supplement: Supplementary file 1 [file DataSheet1.pdf]

Supplementary Material

1 Search Strategy

Searches were conducted across seven electronic databases from inception to March 2026, with no language restriction. The database-specific search strings are reproduced below. Boolean syntax was adapted to the requirements of each platform; minor formatting was standardized for presentation without altering the underlying search logic.

PubMed

(“Tetris” OR “visuospatial task” OR “imagery-competing task” OR “imagery competing task” OR “visuospatial interference” OR “visuospatial cognitive task” OR “cognitive task intervention” OR “brief cognitive task” OR “dual-task intervention” OR “dual task intervention” OR “ICTI” OR “mental rotation” OR “memory reactivation” OR “memory reminder” OR “reconsolidation intervention” OR “visuospatial game”) AND (“intrusive memor*” OR “intrusion*” OR “flashback*” OR “re-experiencing” OR “reexperiencing” OR “involuntary memor*” OR “unwanted memor*” OR “PTSD” OR “post-traumatic stress” OR “posttraumatic stress” OR “acute stress disorder” OR “trauma memor*”) AND (“randomized controlled trial”[pt] OR “randomised controlled trial” OR “RCT” OR “random*” OR “Bayesian adaptive” OR “clinical trial”[pt])

Scopus

“intrusive memories” OR “flashbacks” “Tetris” OR “visuospatial task” OR “imagery competing task” OR “ICTI” randomized trauma

Web of Science

TS=((“Tetris” AND (“trauma” OR “PTSD” OR “intrusive” OR “flashback”)) OR “imagery-competing task*” OR “imagery competing task*” OR “visuospatial cognitive task*” OR “brief cognitive task*” OR “ICTI” OR “visuospatial game*”) AND TS=(“intrusive memor*” OR “flashback*” OR “re-experiencing” OR “reexperiencing” OR “involuntary memor*” OR “unwanted memor*” OR “post-traumatic stress” OR “posttraumatic stress” OR “acute stress disorder”) AND TS=(“randomized controlled trial” OR “randomised controlled trial” OR “RCT” OR “Bayesian adaptive trial”)

Psychology Collection (ProQuest Psychology Journals)

AB(Tetris OR “imagery-competing task” OR “imagery competing task” OR “visuospatial cognitive task” OR “brief cognitive task” OR ICTI OR “visuospatial game” OR “visuospatial task” OR “visuospatial interference”) AND AB(intrusive memor* OR flashback* OR post-traumatic stress OR posttraumatic stress OR acute stress disorder OR re-experiencing OR involuntary memor*)

MEDLINE

abstract(“intrusive memories” OR “intrusive memory” OR “flashbacks” OR “flashback” OR “re-experiencing” OR “reexperiencing” OR “involuntary memories” OR “involuntary memory” OR “unwanted memories” OR “post-traumatic stress” OR “posttraumatic stress” OR “acute stress disorder”) AND abstract(“Tetris” OR “imagery-competing task” OR “imagery competing task” OR “visuospatial cognitive task” OR “brief cognitive task” OR “visuospatial task” OR “visuospatial interference” OR “ICTI” OR “visuospatial game”) AND abstract(“randomized controlled trial” OR “randomised controlled trial” OR “RCT” OR “random allocation” OR “randomly assigned” OR “controlled trial” OR “clinical trial”)

Google Scholar

“intrusive memories” OR “flashbacks” “Tetris” OR “visuospatial task” OR “imagery competing task” OR “ICTI” randomized trauma

Google Scholar was searched using the targeted string above. The first 100 results were screened by title and abstract; 98 records identified as potentially eligible were added manually to the screening database.

Ovid

“intrusive memories” OR “flashbacks” “Tetris” OR “visuospatial task” OR “imagery competing task” OR “ICTI” randomized trauma

2 Supplementary Methods: Metric Sensitivity Analysis

To assess whether the choice of effect-size metric influenced the primary conclusion, two separate random-effects meta-analyses (DerSimonian–Laird) were performed using different metrics on the same outcome (intrusive memory frequency per week).

Pool A (Hedges' g). Three studies that reported raw means and standard deviations were converted to Hedges' g and pooled using the inverse-variance method.

Pool B (log-IRR). Two studies that reported incidence rate ratios were converted to log-incidence rate ratios with their standard errors and pooled using the generic inverse-variance method.

3 Supplementary Results

Standardized mean difference (Hedges' g). The pooled Hedges' g was −0.489 (95% CI −0.798 to −0.180), favouring the ICTI intervention. Heterogeneity was negligible (I^2 = 0%, p = 0.856). The forest plot is shown in Supplementary Figure 1.

Log incidence rate ratio (log-IRR). The pooled log-IRR was −1.187 (95% CI −1.589 to −0.786), corresponding to an IRR of 0.31 (95% CI 0.20 to 0.46). Heterogeneity was absent (I^2 = 0%, p = 0.936). The forest plot is shown in Supplementary Figure 2.

Conclusion. Both metric sensitivity analyses produced effect estimates favouring ICTI, with narrow confidence intervals and no heterogeneity. These results confirm that the primary finding (pooled raw mean difference = −6.98, 95% CI −9.84 to −4.13) is robust to the choice of effect-size metric.

4 Supplementary Figures

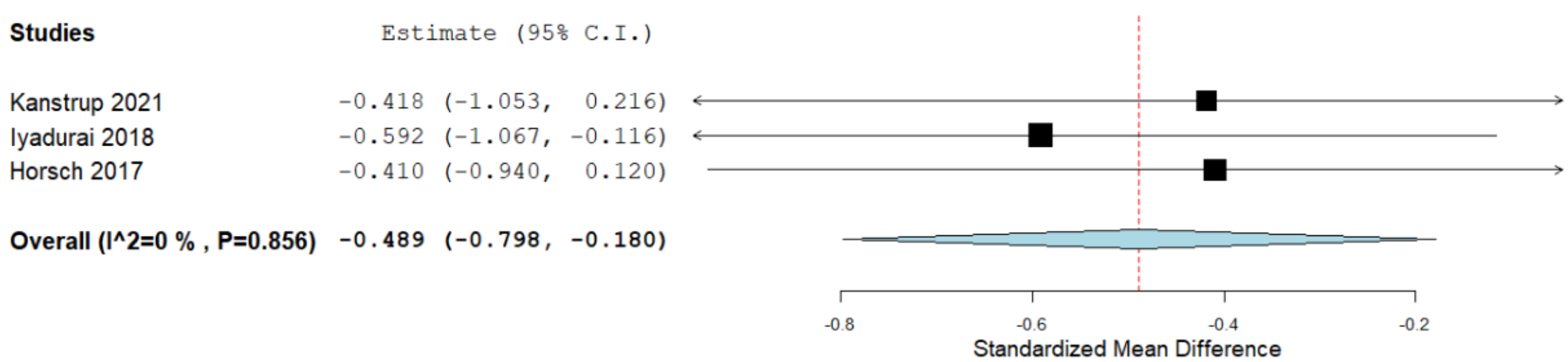

Supplementary Figure 1. Forest plot of the metric sensitivity analysis for intrusive memory frequency, Pool A (Hedges' g). Three parallel-group randomised controlled trials (Kanstrup 2021, Iyadurai 2018, Horsch 2017) reporting raw means and standard deviations were converted to Hedges' g and pooled using a random-effects (DerSimonian–Laird) inverse-variance model. The pooled standardized mean difference was −0.489 (95% CI −0.798 to −0.180), favouring the intervention, with no observed heterogeneity (I^2 = 0%, p = 0.856). Negative values indicate fewer intrusive memories in the ICTI arm relative to control.

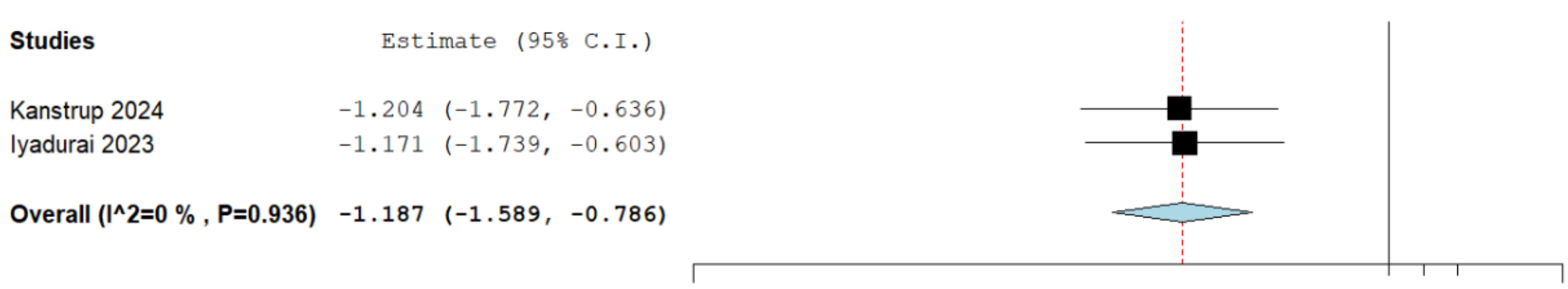

Supplementary Figure 2. Forest plot of the metric sensitivity analysis for intrusive memory frequency, Pool B (log incidence rate ratio). Two parallel-group randomised controlled trials (Kanstrup 2024,

Iyadurai 2023) reporting incidence rate ratios were converted to log-incidence rate ratios with their standard errors and pooled using a random-effects (DerSimonian–Laird) generic inverse-variance model. The pooled log-IRR was −1.187 (95% CI −1.589 to −0.786), corresponding to an incidence rate ratio of 0.31 (95% CI 0.20 to 0.46), with no observed heterogeneity ($I^2 = 0\%$, $p = 0.936$). Negative log-IRR values indicate fewer intrusive memories in the ICTI arm relative to control.

5 Supplementary Tables

Supplementary Table 1. Adverse events reported in the included studies. AE, adverse event; SAE, serious adverse event; ICTI, imagery-competing task intervention; VR, virtual reality. A dash (–) indicates that the item was not applicable or not reported.

First author, year	AEs reported	ICTI arm	Control arm	Study-related SAEs	Notes
Beckenstrom 2026	Yes	51 AEs in 37% of participants	Similar	None related to ICTI	2 AEs due to diary burden
Kanstrup 2024	Yes	64 AEs (no SAEs)	119 AEs (p = 0.0052)	None	Control arm reported significantly more AEs
Iyadurai 2023	Yes	None related to intervention	None	None	–
Kanstrup 2021	Yes	None reported	None	None	–
Horsch 2017	Yes	No harmful effects	No harmful effects	None	–
Freedman 2020	Yes	Low nausea from VR (M = 2.42, SD = 2.56)	Not applicable	None	–
Kehyayan 2024	Yes	No adverse events reported	No adverse events	None	–
Deforges 2023	Not reported	–	–	–	–
Hardarson 2024	Not reported	–	–	–	–
Badawi 2021	Not reported	–	–	–	–
Holmes 2017	Not reported	–	–	–	–

Supplementary Table 2. Baseline intrusive memory frequency and PTSD symptoms by arm, with baseline equivalence. ICTI, imagery-competing task intervention; IQR, interquartile range; SD, standard deviation; PTSD, post-traumatic stress disorder; PCL-5, PTSD Checklist for DSM-5; IES-R, Impact of Event Scale–Revised; ASDS, Acute Stress Disorder Scale; PSS, Posttraumatic Symptom Scale; ns, not significant; N/A, not applicable. A dash (–) indicates not reported.

First author, year	Baseline intrusions – ICTI	Baseline intrusions – Control	Baseline PTSD measure	Baseline PTSD – ICTI	Baseline PTSD – Control	Baseline equivalence (Y/N)
Beckenstrom 2026	Median 11.5 (IQR 9.0–18.0)	Median 9.0 (IQR 6.5–16.0)	PCL-5 (20-item)	Not reported separately	Not reported separately	Yes (p = 0.12, ANOVA)
Iyadurai 2018	Not reported (diary post-trauma only)	Not reported	IES-R (intrusion)	Not assessed at baseline	Not assessed at baseline	Yes (no significant differences)
Kanstrup 2024	Mean 21.79 (SD 21.32); Median 16.0	Mean 19.48 (SD 16.50); Median 14.0	PCL-5 (8-item)	Not reported by arm	Not reported by arm	Yes (comparable)
Iyadurai 2023	Mean 17.08 (SD 18.5); Median 14.5 (IQR 10–21.5)	Mean 15.84 (SD 13.61); Median 14.0 (IQR 8–19)	PCL-5 (4-item)	8.71 (SD 3.64)	8.00 (SD 3.27)	Yes
Deforges 2023	Not assessed	Not assessed	PCL-5	Not assessed	Not assessed	Yes (groups well represented)
Kanstrup 2021	Not reported	Not reported	Not assessed	N/A	N/A	Yes

First author, year	Baseline intrusions – ICTI	Baseline intrusions – Control	Baseline PTSD measure	Baseline PTSD – ICTI	Baseline PTSD – Control	Baseline equivalence (Y/N)
Horsch 2017	Not reported (diary post-trauma only)	Not reported	ASDS	19.32 (SD 4.58)	21.58 (SD 8.06)	Yes (all p ns)
Freedman 2020	PSS intrusion: mean 0.94 (SD 1.43)	PSS intrusion: mean 0.76 (SD 1.46)	PSS total	2.39 (SD 2.96)	2.05 (SD 3.15)	Yes (U = 579, p = 0.89)
Kehyayan 2024	Mean 2.02 (SD 1.92) per day (Tetris memories)	Mean 2.12 (SD 1.89) per day (Wiki memories)	Not measured as outcome	N/A	N/A	Yes (baseline frequencies comparable)
